# Novel mediator in anaphylaxis: decreased levels of miR-375-3p in serum and within extracellular vesicles of patients

**DOI:** 10.3389/fimmu.2023.1209874

**Published:** 2023-10-30

**Authors:** Emilio Nuñez-Borque, Sergio Fernandez-Bravo, Pablo Rodríguez Del Rio, Lucia Palacio-García, Angela Di Giannatale, Virginia Di Paolo, Angela Galardi, Marta Colletti, Luisa Pascucci, Jaime Tome-Amat, Javier Cuesta-Herranz, María Dolores Ibañez-Sandin, José Julio Laguna, Alberto Benito-Martin, Vanesa Esteban

**Affiliations:** ^1^ Department of Allergy and Immunology, IIS-Fundación Jiménez Díaz, Universidad Autónoma de Madrid (UAM), Madrid, Spain; ^2^ Allergy Department, Hospital Infantil Universitario Niño Jesús, Fundación Hospital Niño Jesús (HNJ), Instituto de Investigación del Hospital de La Princesa (IIS-P), Madrid, Spain; ^3^ Department of Pediatric Onco-Hematology and Cell and Gene Therapy, Bambino Gesù Children’s Hospital, Istituti di Ricovero e Cura a Carattere Scientifico (IRCCS), Rome, Italy; ^4^ Department of Veterinary Medicine, University of Perugia, Perugia, Italy; ^5^ Centro de Biotecnología y Genómica de Plantas, Universidad Politécnica de Madrid-Instituto Nacional de Investigación y Tecnología Agraria y Alimentaria (UPM-INIA), Universidad Politécnica de Madrid, Madrid, Spain; ^6^ Department of Allergy. Fundación Jiménez Díaz University Hospital, Universidad Autónoma de Madrid (UAM), Madrid, Spain; ^7^ Allergy Unit, Allergo-Anaesthesia Unit, Cruz Roja Central Hospital, Villanueva de la Cañada, Madrid, Spain; ^8^ Faculty of Medicine and Biomedicine, Universidad Alfonso X el Sabio (UAX), Madrid, Spain

**Keywords:** anaphylaxis, biomarker, endothelial permeability, extracellular vesicles, miRNA

## Abstract

**Introduction:**

Anaphylaxis is among the most severe manifestations of allergic disorders, but its molecular basis remains largely unknown and reliable diagnostic markers are not currently available. MicroRNAs (miRNAs) regulate several pathophysiological processes and have been proposed as non-invasive biomarkers. Therefore, this study aims to evaluate their involvement in anaphylactic reaction and their value as biomarkers.

**Methods:**

Acute (anaphylaxis) and baseline (control) serum samples from 67 patients with anaphylaxis were studied. Among them, 35 were adults with drug-induced anaphylaxis, 13 adults with food-induced anaphylaxis and 19 children with food-induced anaphylaxis. The circulating serum miRNAs profile was characterized by next-generation sequencing (NGS). For this purpose, acute and baseline samples from 5 adults with drug-induced anaphylaxis were used. RNA was extracted, retrotranscribed, sequenced and the readings obtained were mapped to the human database miRBase_20. In addition, a system biology analysis (SBA) was performed with its target genes and revealed pathways related to anaphylactic mediators signaling. Moreover, functional and molecular endothelial permeability assays were conducted with miR-375-3p-transfected cells in response to cAMP.

**Results:**

A total of 334 miRNAs were identified, of which 21 were significant differentially expressed between both phases. Extracellular vesicles (EVs) were characterized by Western blot, electron microscopy and NanoSight. A decrease of miR-375-3p levels was determined by qPCR in both serum and EVs of patients with anaphylaxis (****p<.0001). Precisely, the decrease of miR-375-3p correlated with the increase of two inflammatory cytokines: monocyte chemoattractant protein-1 (MCP-1) and granulocyte macrophage colony-stimulating factor (GM-CSF). On the other hand, functional and molecular data obtained showed that miR-375-3p partially blocked the endothelial barrier maintenance and stabilization by disassembly of cell-cell junctions exhibiting low Rac1-Cdc42 levels.

**Discussion:**

These findings demonstrate a differential serum profile of circulating miRNAs in patients with anaphylaxis and exhibit the miR-375-3p modulation in serum and EVs during drug- and food-mediated anaphylactic reactions. Furthermore, the *in silico* and *in vitro* studies show a negative role for miR-375-3p/Rac1-Cdc42 in the endothelial barrier stability.

## Graphical Abstract

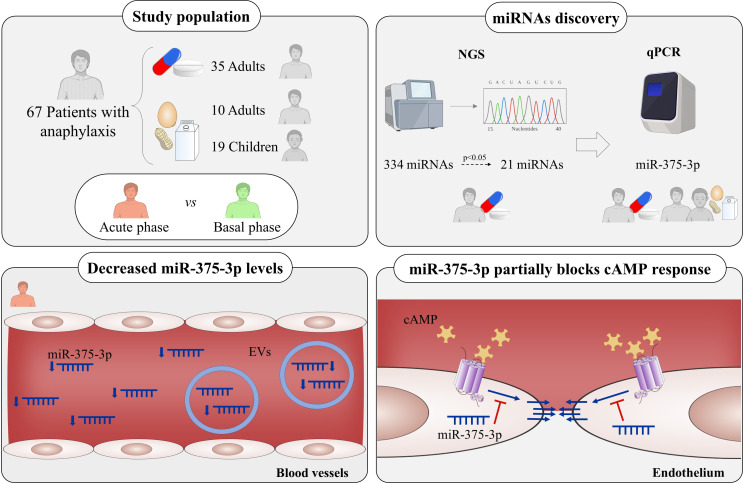

## Introduction

Anaphylaxis is a potentially life-threatening hypersensitivity reaction and one of the most severe manifestation of allergic disorders (1). The major triggers of this pathological event include drugs, foods and Hymenoptera venoms. Specifically, drugs are the main allergens provoking anaphylaxis in adults, while foods are the most common cause of reaction in children (2, 3).

The diagnosis of anaphylaxis is based on clinical symptoms, but sometimes remains elusive as some manifestations are common to many other pathologies (4), and clinical criteria are not fully consistent across different expert consensus (5). Currently, the main biomarker used in clinical practice to complement diagnosis is serum tryptase. However, this molecule is not increased in many cases, with poor reliability in mild cases where it is most needed to aid in the differential diagnosis process (6). Therefore, it of utmost relevance to find novel and reliable molecular markers that allows us to enlarge the diagnosis of this pathological event.

Mechanistically, anaphylaxis is mainly produced by IgE-mediated activation of reaction’s effector cells, mast cells and basophils (7). However, other IgE-independent molecular processes and cells are involved in this pathological event (8). Overall, there is a release of mediators that gives rise to signs and symptoms of anaphylaxis (9). Among them, the cutaneous ones are the most frequent, although the respiratory and cardiovascular systems mark the development of the most severe cases (10). In turn, vessels´ endothelium is the main component affected during anaphylaxis. This structure, composed of an extensive monolayer of endothelial cells (ECs), forms a physical barrier between blood and tissues allowing selective transport of molecules (11). Mediators released by effector cells act on ECs destabilizing the monolayer and leading to increased endothelial permeability and fluid extravasation through mechanisms that mainly regulates disruption of cell junctions and cytoskeletal disability (12).

MicroRNAs (miRNAs) are small non-coding RNA molecules (~22 nucleotides) that can be found circulating in serum and other body fluids associated with protein complexes or within extracellular vesicles (EVs) (13). In addition, due to their stability and their ability to mirror the pathophysiological state of the patient, they have been proposed as promising non-invasive biomarkers (14). miRNA transcripts are processed, resulting in a mature duplex miRNA which strands separate giving rise to the RNA-induced silencing complex (RISC) that regulates the translation of messenger RNAs (mRNAs) (15). miRNAs modulate many physiological functions (13, 16). Specifically, those transported by EVs may mediate communication between different tissues (13). Therefore, miRNAs levels changes are relevant in a wide range of allergic diseases (16). Precisely, our group reported the increase of miR-21-3p and miR-487b-3p levels during the acute phase of anaphylaxis in a study including 19 children with food-induced reactions (17). Moreover, elevated values of miR-451a have also been described in 16 adults with food- and Hymenoptera venom-mediated anaphylaxis (18). However, miRNAs levels have never been characterized in adults with drug-mediated reactions.

Therefore, this study aims to determine the serum circulating miRNA profile in adults with drug-anaphylactic mediated reactions, to evaluate their capacity as diagnostic biomarkers and to investigate the possible involvement of these molecules in the underlying molecular basis of the reaction.

## Materials and methods

### Study population

Initial population included 204 paired sera from 102 patients presenting anaphylaxis and recruited from three Spanish hospitals (Fundación Jiménez Díaz University Hospital, Cruz Roja Central Hospital and Niño Jesús University Children’s Hospital). However, 35 of them were discarded due to the hemolysis of their samples.

Anaphylaxis diagnosis was confirmed by an allergist in agreement with the definition of anaphylaxis established by the “National Institute of Allergy and Infectious Disease and Food Allergy and Anaphylaxis Network” (19). In addition, from each patient were recorder their gender, age, the trigger of the reaction and if they were treated before or after obtaining the acute sample. Furthermore, their clinical signs and symptoms were also collected and, based on these, the severity of the reaction was determined according to the criteria established by Brown (20).

The study was approved by the Ethics Committee (CEIm FJD, PIC057-19), authors adhered to the declaration of Helsinki and all patients were included after giving informed consent by the donors or their relatives. Inclusion criteria were acceptance to participate in the study and an objective diagnosis of the reaction. Exclusion criteria were the presence of a blood-borne disease and/or any psychic or psychological diseases that would prevent acceptance for the study.

### Sample collection

Serum samples were collected from each patient under two conditions: during acute phase (anaphylaxis) and at basal phase (control), at least 14 days after the reaction. Considering the heterogeneity of participants, the acute sample data was normalized by calculating the increase or decrease ratio upon baseline individual sample values (ratio acute/basal).

All samples were obtained from patients who developed anaphylaxis accidentally. If the allergic reaction occurred accidentally, acute phases were collected in emergency departments. On the other hand, if the hypersensitivity reaction was provoked by a controlled challenge test, sera were obtained in allergy units.

For serum processing, tubes with a separator gel (BD Vacutainer) were used. Once the peripheral blood was obtained, it was centrifuged at 1,200 g for 10 minutes at 4°C. Afterwards, the serum was aliquoted in 0.5 ml and stored at -80°C until its use in each of the experiments.

### Profile of serum circulating miRNAs

The serum circulating miRNAs profile was determined by Next Generation Sequencing (NGS) at Qiagen Genomic Services, as previously described by Nuñez-Borque et al. (17, 21). For the analysis, acute and baseline samples from 5 adult patients with drug anaphylaxis were tested in the same batch.

RNA was extracted from 200 μl of serum using the miRNeasy Serum/Plasma Kit (Qiagen). Moreover, 52 exogenous sequences were included to confirm the correct isolation of miRNAs. In turn, libraries were performed with the QIAseq miRNA Library Kit (Qiagen). For this purpose, RNA was retrotranscribed to copy DNA (cDNA), amplified by PCR (22 cycles) and purified from samples. Libraries were carried out by a gel-free system through the addition of Unique Molecular Identifiers (UMIs) and were specific for small RNA species ranging from 15-40 nucleotides. Quality controls were determined using the Bioanalyzer 2100 or the TapeStation 4200 (Agilent Technologies). In turn, miRNAs data were normalized to equimolar ratios depending on the quality of the library, the quality of the inserts and the concentration measurements. Finally, libraries were quantified by quantitative PCR (qPCR) and sequenced on an Illumina NextSeq500 instrument. Raw data files (FASTQ) were generated for each sample using the bcl2fastq program (Illumina Inc.) and Cutadapt (1.11) software was utilized to remove adapter sequences and collapse the reads by UMIs. Readings were mapped against the miRbase_20 database using the Bowtie2 program (2.2.2) and no errors or more than 1 mismatch were allowed.

### Serum miRNAs extraction, retrotranscription and quantification

To the validation step, paired samples from each patient were always analyzed simultaneously. Serum miRNAs were extracted using the miRNeasy Serum/Plasma Advanced kit (Qiagen). For each sample, 300 μl of serum were used and added 90 μl of lysis buffer and 1 μl of UniSp2/4/5 mix (miRCURY LNA RNA Spike-in kit, Qiagen), synthetic miRNAs that serve as a quality control of the technique. Following this, samples were incubated for 3 minutes at room temperature, 30 μl of precipitation buffer were added, and a new 3-minute incubation at room temperature was performed. Tubes were centrifuged at 12,000 g for 3 minutes, supernatants were mixed with an equal volume of isopropanol and transferred to a specific column where RNA was retained. Subsequently, three washes were performed and 15 μl of RNAase-free water was added to the column, which was incubated for 1 minute at room temperature and centrifuged for 1 minute to elute the isolated RNA.

Total RNA was retrotranscribed using the miRCURY LNA RT kit (Qiagen). For this purpose, 2 μl of the reaction-specific buffer, 4.5 μl of RNAase-free water, 1 μl of the enzyme mixture, 2 μl of the RNA previously obtained in the extraction and 0.5 μl of UniSp6, a synthetic miRNA used as a quality control of the reverse transcription, were included for each sample. Once the mix was done, samples were placed in the PTC-100 Thermal Cycler (MJ research, Inc) where retrotranscription was carried out.

Finally, miRNAs quantification (miRCURY LNA SYBR Green PCR kit, Qiagen) was performed by quantitative PCR (qPCR) with specific primers (Qiagen) in the LightCycle96 Real Time PCR System (Roche Life Science). Samples from each patient were always measured at the same time and in the same batch. UniSp2, UniSp4, UniSp5 and UniSp6 were used as exogenous controls of the extraction and reverse transcription, while miR-451a and miR-23a-3p were measured as endogenous controls of sample hemolysis (22, 23). Moreover, miR-30e-5p was chosen as endogenous control for the normalization of the target miRNAs, as suggested by the NGS according to the levels of all molecules identified. Data obtained were analyzed using the 2^-ΔΔCT^ method (24).

### Purification of extracellular vesicles

The purification of EVs from serum was performed using the miRCURY Exosome isolation Kit – Serum and Plasma (Qiagen) according to Colletti et al. (25). For this purpose, 500 μl of serum were centrifuged at 12,000 g for 20 minutes at 10°C. Subsequently, 200 μl of the precipitate buffer were added to the supernatant and the mix was incubated for 3 hours at 4°C. After this time, samples were centrifuged at 1,500 g for 30 minutes and supernatants were removed. Finally, EVs were collected adding 270 μl of the resuspension buffer.

Suitable isolation and characterization of EVs was determined by Western blot (WB), nanoparticle tracking analysis (NTA) and electron microscopy (EM) as previously described (26) and following MISEV2018 guidelines (27). Subsequently, the extraction, retrotranscription and quantification of miRNAs contained in EVs were carried out as previously described in the section “Serum miRNAs extraction, retrotranscription and quantification”. Paired samples from each patient were always analyzed simultaneously.

### Western blot

Three bona fide markers (CD9, CD63 and TSG101) of EVs were characterized by WB. Proteins were separated by electrophoresis on sodium dodecyl sulfate polyacrylamide gels (SDS-PAGE) at 12.5% using the PowerPac Basic Power Supply section (BioRad). Subsequently, proteins were transferred to a nitrocellulose membrane (BioRad) using the Trans-Blot Turbo Transfer system (BioRad). Non-specific membrane junctions were blocked by applying a solution of phosphate buffered saline (PBS) with 0.1% Tween (Sigma) and 5% milk. After this, the membrane was incubated overnight with the corresponding primary antibodies diluted 1:500 in all cases: anti-CD9 (Invitrogen), anti-CD63 (Invitrogen) and anti-TSG101 (Abcam). For signal detection, the membrane was incubated for 1 hour with a rabbit anti-mouse (RAM) peroxidase-conjugated secondary antibody (Jackson Laboratory) diluted at 1:5,000 in 0.05% PBS-Tween solution with 3% milk. Lastly, proteins were detected in the Amersham Imager 600 system (GE Healthcare) using the chemiluminescent ECL Prime Western Blotting Detection Reagent (Thermo Scientific).

### Electron microscopy

For the EVs visualization by EM, 5 μl of the previously purified particles were fixed for 24 hours at 4°C using 50 μl of PBS with 2% paraformaldehyde (PFA, Sigma). After this, EVs were adsorbed on copper/carbon grids (Fedelco) for 3 minutes, washed with water for 1 minute and counterstained with 1% uranyl acetate for 30 seconds. Then, samples were observed using a JEOL 1010 transmission EM (JEOL) and employing a voltage of 100 kV. The analysis and image processing were carried out with the Soft Imaging Viewer software.

### Nanoparticle tracking analysis

NTA was performed at the Spanish National Cancer Research Center (CNIO, Madrid) with the NS500 nanoparticle characterization system (NanoSight) equipped with a blue laser (405 nm) and diluting samples 1:500.

### System biology analysis

Systems Biology Analysis (SBA) of miR-375-3p was performed *in silico* using all its target genes with more than 50 target score (269 genes) in the miRDB database (http://mirdb.org/) (28). All genes were mapped against their corresponding gene ontology (GO) in the Ingenuity Pathway Knowledge Base using the Ingenuity Pathway Analysis (IPA) software (Qiagen). This program provided different functional categories: “ingenuity canonical pathways”, “upstream regulators”, “disease and disorders”, “network” and “tox lists”. In addition, it evaluated the significance using a one-tailed Fisher’s test. Among the different functional categories obtained, “disease and disorders” and “ingenuity canonical pathways” were the only one considered.

### Quantification of serum cytokines

Serum cytokine levels were determined using the multiplex assay RayPlex Human Cytokine Storm Array 1 Kit (RayBiotech). For this purpose, acute and basal samples from 40 patients with anaphylaxis were studied. Dilution of the samples and standards was performed according to the manufacture indications. A panel of 25 mediators involved in the inflammatory process was tested: basic Fibroblast growth factor (bFGF), Eotaxin, Granulocyte colony-stimulating factor (G-CSF), Granulocyte macrophage colony-stimulating factor (GM-CSF), Interferon-γ (IFNγ), Interleucin-10 (IL-10), IL-12p70, IL-13, IL-15, IL-17A, IL-1β, IL-1RA, IL-2, IL-4, IL-5, IL-6, IL-7, IL-8, Monocyte chemoattractant protein-1 (MCP-1), Macrophage inflammatory protein-1α (MIP-1α), MIP-1β, Platelet-derived growth factor-BB (PDGF-BB), RANTES, Tumor necrosis factor-α (TNFα), Vascular endothelial growth factor (VEGF). Mean fluorescent intensities of the samples and standards were determined in a paired manner using a BD FACSCanto II flow cytometer (Becton Dickinson).

### Transfection of miRNA

Human dermal microvascular ECs (HMVEC-D) were acquired from Lonza (CC-2543) and maintained in EGM-2MV BulletKit medium, as previously described (29). First, HMVEC-D were seeded and allowed to grow for 24 hours to reach 70-80% confluence. Next day, transfection was conducted incubating cells with Opti-MEM medium (Thermo Scientific) and using Transit-X2 reagent and mimics (50 mM) of the miR-375-3p (Qiagen). In addition, fluorescently labeled mimic scrambles (50 mM) were applied as controls of the technique (Qiagen). Subsequently, after 72 hours, molecular and functional studies were performed in HMVEC-D. In turn, the correct development of the transfection was confirmed by qPCR and confocal microscopy.

### miRNA extraction from endothelial cells

Extraction of miRNAs from HMVEC-D was carried out using the MasterPure Complete DNA & RNA Purification Kit (Lucigen). Cells were lysed by adding 300 μl of lysis buffer and 1 μl of Proteinase K. The lysate obtained was collected and incubated at 65°C for 15 minutes. Then, samples were incubated at 4°C for 5 minutes and 150 μl of precipitation buffer were added. Subsequently, each sample was centrifuged at 10,000 g for 10 minutes and supernatants were collected. An equal volume of isopropanol was added, and mixtures were centrifuged again. Next, to remove the remaining DNA, the precipitate was resuspended in 200 μl of the DNA nuclease buffer and 5 μl of DNA nuclease I. Consecutively, samples were incubated at 37°C for 30 minutes and 200 μl of the lysis and precipitation buffers were added. Then, samples were incubated at 4°C for 5 minutes and centrifuged at 10,000 g for 10 minutes at 4°C. After this, an equal volume of isopropanol was added, and samples were centrifuged again. Finally, the RNA extracted was resuspended in 20 μl of trisaminomethane buffer (TE) and 1 μl of RNAse inhibitor. Finally, reverse transcription and miRNAs quantification were performed as previously described in the section “Serum miRNAs extraction, retrotranscription and quantification”.

### Protein extraction and quantification from endothelial cells

Cells were seeded and transfected in P6 plates (Corning) as previously described in the “Transfection of miRNA” section. 30 µM cyclic AMP (cAMP) was used to stabilize the endothelial barrier during 30 minutes. Once the incubation time was over, proteins were extracted by adding 100 μl of lysis buffer supplemented with 5 mM protease inhibitor (Sigma), 1 mM phenylmethylsulfonyl fluoride (PMSF, Sigma) and 5 mM dithiothreitol (DTT, BioRad). Cells were detached using a scraper and the lysates obtained were kept in agitation for 15 minutes at 4°C. After this, they were centrifuged for 15 minutes at 12,000 rpm at 4°C and the supernatants, which contained the proteins, were stored at -20°C until use. Rac1-CDC42 levels were analyzed by WB following the protocol previously described in the “Western Blot” section. Membranes were incubated overnight with primary antibody anti-Rac1-Cdc42 (Cell Signaling) diluted 1:500. For signal detection, the membrane was incubated for one hour with a goat anti-rabbit (GAR) peroxidase-conjugated secondary antibody (Jackson Laboratory) diluted at 1:5,000 in 0.05% PBS-Tween soluction with 3% milk. Likewise, an anti-β-actin antibody (Santa Cruz) was used to detect β-actin as a housekeeping protein diluted at 1:500 while the RAM secondary antibody was diluted 1:5,000. Bands intensity was quantified using the ImageJ software and normalization to the amount of total protein loaded was calculated using the ratio of the Rac1-Cdc42/β-actin signal.

### Endothelial permeability assay

The endothelial barrier integrity was assessed by measuring the trans-endothelial electrical resistance (TEER) value of the monolayer through an EndOhm chamber (WPI) using 24-well Transwell (TWs) cell culture inserts (26, 29). For determining the effect of miR-375-3p in a cAMP-stabilized endothelial system, HMVEC-Ds were transfected on TW. Four different HMVEC-D systems were registered and analyzed: non transfected, incubated with Transit-X2 alone, transfected with the scramble, as control, and transfected with the miR-375-3p mimic (50 mM). All these systems were evaluated in presence of cAMP and TEER measures were obtained at different times (10, 30, 45, 60, 90, 120 and 180 minutes).

### Confocal microscopy

For confocal microscopy HMVEC-D were seeded in P8 chambers (IBIDI). Cells were fixed with 4% PFA for 15 minutes and permeabilized with 0.1% Triton (Sigma) for 5 minutes at 4°C. After this, non-specific junctions were blocked by adding a PBS solution with 2% BSA for 20 minutes. Cells were incubated overnight with an anti-VE-cadherin antibody (Cell Signaling) diluted 1:400 in a solution of PBS with 2% BSA. The next day, cells were incubated for 1 hour with a donkey anti-rabbit Alexa Fluor 647-conjugated secondary antibody (Invitrogen). Then, actin filaments were red stained by incubating HMVEC-D for 30 minutes with Texas-Red-X phalloidin reagent (Invitrogen) at a concentration of 1:40 in a solution of PBS with 2% BSA. Subsequently, a second 10-minutes incubation was carried out with a solution of DAPI (Sigma) at a concentration of 1:1,000 in distilled water to stain the nuclei blue. Lastly, the slides were assembled using the FlourSave Reagent (Calbiochem) and visualized on the LSM 700 inverted confocal microscope (Carl Zeiss). Transfection was confirmed by visualization of the fluorescent scramble (green) inside ECs.

### Statistical analysis

Data obtained from NGS were normalized and analyzed with the statistic software R 3.5.3. The principal component analysis (PCA) and the heat map were carried out on the ClustVis website (https://biit.cs.ut.ee/clustvis/) (30). Graphical representation and statistical evaluation were performed by using the Graph Pad Prism 8 software (La Jolla, CA, USA).

Qualitative variables were described as frequency and percentage. However, in quantitative variables, data distribution was always first assessed by normality tests. When data presented a normal distribution, parametric analyses were used, and the graphs were represented by the mean ± standard error of the mean (SEM). On the other hand, when the data did not follow a normal distribution, non-parametric tests were used, and the graphs showed the median ± interquartile range (IQR). Differences were considered significant at level of *p*<.05.

NGS analysis were performed with the Prostar package (http://live.prostar-proteomics.org/) distributed by Bioconductor and implemented in R (R Core Team, 2019). Abundance data were transformed (log_2_) using R 3.5.3 software to obtain a symmetrical distribution. Moreover, all miRNAs not detected in at least two patients were discarded. The matrices were normalized with the Cyclic Loess method to reduce systemic variances (31). Finally, Student’s paired t-test was used to determine the significant differences between both conditions (acute vs basal) (32).

For the validation stage and measurements of miRNAs within EVs, data did not follow a normal distribution, so the two-tailed Wilcoxon matched-pairs non-parametric test was used to determine the significant differences between acute and basal phases. In turn, for the characterization of EVs purified from serum, non-parametric tests were used and both size and concentration were analysed with the Wilcoxon matched-pairs test. On the other hand, the study of serum cytokines was carried out using the Wilcoxon matched-pairs test in all cases. In turn, comparison of their levels with those of miR-375-3p was performed using Spearman’s non-parametric correlation test. Moreover, data obtained from *in vitro* assays followed a normal distribution and, consequently, analyses were performed using the paired t-test or 2 way ANOVA.

## Results

### Characteristic of the patients´ cohorts

The studied cohort included 67 patients with anaphylaxis recruited from different Spanish hospitals (**Supplemental Figure 1**). Their clinical characteristics and those of their reactions are shown in **Table 1**. This population aged between 4 and 76 years old and more than half were females. The most frequent symptoms were cutaneous and respiratory, followed by gastrointestinal, mucosal, cardiovascular and central nervous system manifestations. Overall, two thirds of the reactions were classified as moderate (Grade 2), while the remaining ones were categorized as severe (Grade 3). Outstandingly, the frequency of Grade 3 cases was lower in children with food-induced reactions compared to any of the two adult populations (11% vs 43% and 46%, respectively). Finally, even though patient health was always prioritized, a percentage of acute samples were obtained immediately before treatment administration. In these subjects, the most used medications were epinephrine, histamine receptor 1 antagonists and corticosteroids.

**Table 1:** Clinical characteristics of the 67 patients with anaphylaxis used for miRNAs study.

	Patients				*p*-Value		
	Total	Ad	Af	Cf	Ad vs Af	Ad vs Cf	Af vs Cf
**Number**	67	35	13	19	–	–	–
**Sex (female)**	60%	63%	54%	58%	.5801	.7270	.8276
**Age (years) ± SEM**	27.9 ± 2.3	44.6 ± 2.4	33.3 ± 3.2	10.5 ± 0.9	.0104*	.0001*	.0001*
Trigger							
Drug	52%	100%	0%	0%	.0001*	.0001*	>.9999
Food	48%	0%	100%	100%	.0001*	.0001*	>.9999
Symptoms							
Cutaneous	88%	91%	92%	68%	.9240	.1933	.3223
Mucosal	49%	40%	77%	47%	.0228*	.6091	.1005
Gastrointestinal	61%	54%	85%	58%	.0554	.8034	.1164
Respiratory	88%	89%	77%	95%	.3199	.4649	.1434
Neurological	21%	34%	0%	11%	.0142*	.0587	.2405
Cardiovascular	36%	40%	46%	21%	.7081	.1644	.1412
Severity							
Grade 2	66%	57%	54%	89%	.8421	.0140*	.0218*
Grade 3	34%	43%	46%	11%	.8421	.0140*	.0218*
**Treatment†**	72%	80%	77%	53%	.8203	.0359*	.1739
Epinephrine	79%	82%	60%	90%	.1660	.5710	.1346
H1R antagonist	71%	68%	70%	80%	.9037	.4808	.6278
H2R antagonist	21%	21%	30%	10%	.5961	.4373	.2878
Corticosteroids	73%	79%	80%	50%	.9267	.0917	.1769
β2-adrenergic agonist	17%	14%	10%	30%	.7392	.2836	.2878

†Percentage of patients treated prior to acute sample collection; remaining subjects received treatment after obtaining the acute phase serum. * Significant difference between groups (p<.05). Ad, adults with drug-induced anaphylaxis; Af, adults with food-induced anaphylaxis; Cf, children with food-induced anaphylaxis; H1R, histamine receptor 1; H2R, histamine receptor 2.

### Identification of circulating serum miRNA profile in patients with drug-induced anaphylaxis

**Figure 1:**
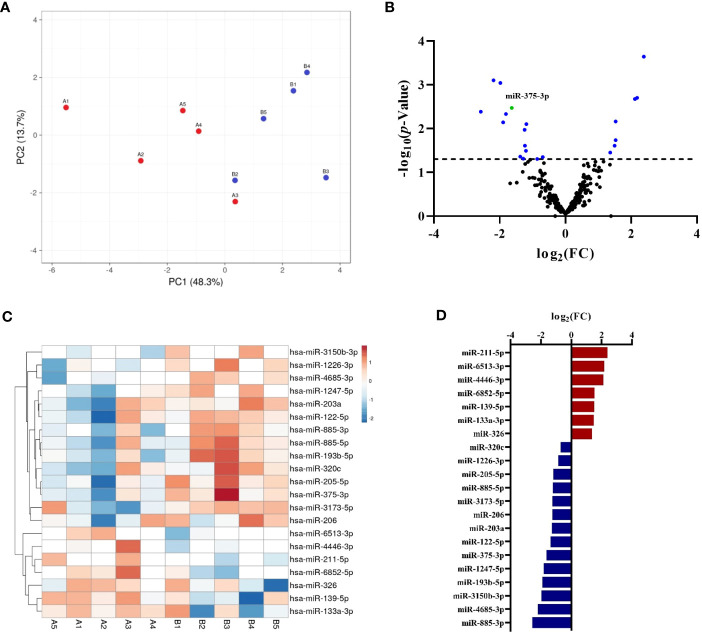
Characterization by NGS of the circulating serum miRNA profile in adults with drug-induced anaphylaxis. **(A)** The PCA showed similarity among the biological replicates and a separation between the acute (A, red points) and basal (B, blue points) conditions. **(B)** Volcano plot exhibits the distribution of total miRNAs identified by NGS. Those statistically significant (*p*<.05) are indicated in blue, whereas miR-375-3p is shown in green. **(C)** Heat map graphically represents the levels of the statistically significant miRNAs in each sample (A: acute phase, B: basal phase). The units represent the transformed (log_2_) and normalized (cyclic Loess method) abundance data obtained by NGS. Red color indicates an increase during the acute phase, whereas blue indicates a decrease. Missing data are represented in white. **(D)** Graphical distribution according to their fold change (FC) of the 14 increased (red) and 7 decreased (blue) miRNAs during the acute phase compared to baseline.

The profile of circulating miRNAs was determined by NGS in an exploratory study carried out in samples from patients with drug anaphylaxis. For this purpose, acute and baseline serum samples were used. The similarity among the biological replicates was verified by the PCA, which exhibited a clear separation between the two stages (**Figure 1A**). In turn, the correct performance of NGS was confirmed through the analysis of different quality controls (**Supplementary Figure 2**).

A total of 334 miRNAs were identified (**Supplementary Table 1**) and after statistical analysis only 21 of them showed significant differences between acute and basal phases (**Figure 1B**; **Supplementary Table 2**). Each sample´s signals dispersion of significant miRNAs is depicted in **Figure 1C**. Among them, 7 were increased during the acute phase, while the remaining 14 were decreased (**Figure 1D**). Interestingly, more than half of these identified miRNAs have been previously described in allergy, although they have never been associated with anaphylaxis (**Supplementary Table 3**).

### Circulating serum levels of miR-375-3p decrease during the acute phase of anaphylaxis compared to baseline

**Figure 2:**
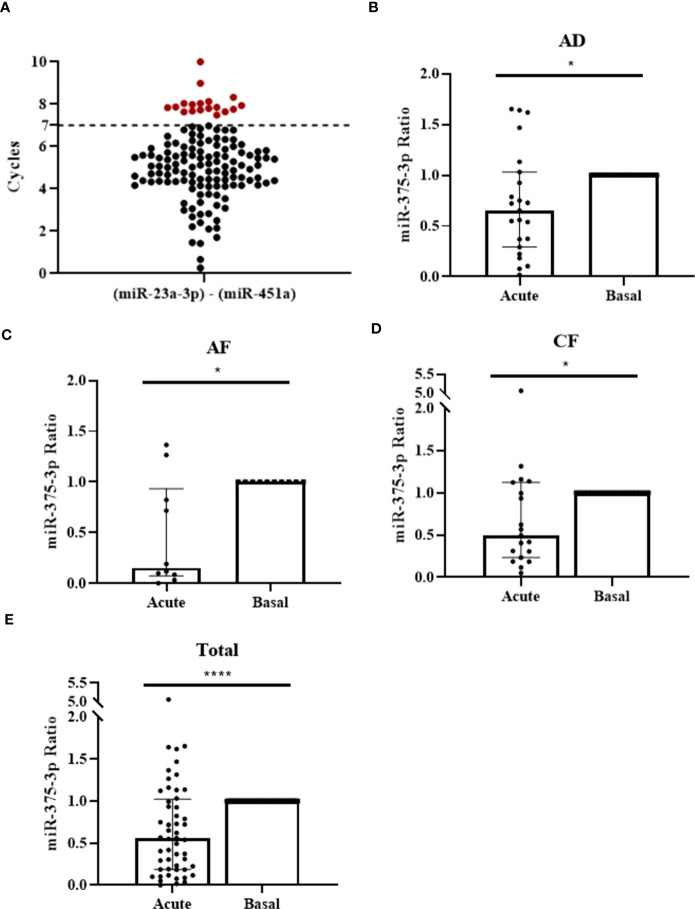
Serum miR-375-3p levels decrease during the reaction in patients with anaphylaxis. **(A)** Measurement of serum hemolysis by the coefficient between the two endogenous controls used: miR-23a-3p and miR-451a. 18 samples exceeded the established threshold (≥7 cycles) and were excluded from the study (red points). **(B)** Serum levels of miR-375-3p decrease in adults with drug-induced anaphylaxis during the acute phase of the reaction compared to baseline (*p=.0196; n: 23). Median ± IQR **(C)** Serum levels of miR-375-3p decrease in adults with food-induced anaphylaxis (*p=.0273; n: 10), **(D)** children with food-induced anaphylaxis (*p=.0181; n: 19) and **(E)** total patients (****p<.0001; n: 52) during the acute phase of the reaction compared to baseline. Median ± IQR.

To evaluate miRNAs capacity as diagnostic biomarkers we extended analysis to 134 samples obtained from 67 patients with anaphylaxis (34 adults with drug-induced reactions and 33 [14 adults and 19 children] with food-induced reactions). 15 patients were discarded from the study because acute and/or basal serum sample presented hemolysis (≥7 cycles) (**Figure 2A**). Therefore, the final cohort included 52 patients with anaphylaxis: 23 adults with drug-induced reactions and 29 (10 adults and 19 children) with food-induced reactions. In all these cases, UniSp2, UniSp4 and UniSp5 were detected approximately 5-7 cycles apart, confirming the correct development of the extraction (**Supplementary Figure 3A**). Furthermore, UniSp6 was amplified around cycle 18 demonstrating that reverse transcription had been successfully carried out (**Supplementary Figure 3B**).

After sample quality control, we choose 6 NGS identified miRNAs (miR-211-5p, miR-139-5p, miR-133a-3p, miR-375-3p, miR-193b-5p, miR-885-3p) to validate their serum level. Statistically significant differences were found exclusively for miR-375-3p, which is decreased during the acute phase of anaphylaxis compared to baseline in adults with drug-induced reactions, as observed in NGS data (FC: -1,63) (**Figure 2B** and **Supplementary Figures 3C–E**). To enlarge its value as a plausible anaphylactic biomarker, we extended the study of miR-375-3p into two cohorts of adults and children food-induced reactions, showing a decrease which was consistent with the results observed in samples from drug-induced reactions (**Figures 2C, D**). Overall, the global analysis of circulating serum levels of miR-375-3p showed a significant reduction during acute phase when compared to baseline in anaphylaxis (**Figure 2E**).

### Levels of miR-375-3p within extracellular vesicles decrease during the acute phase of anaphylaxis compared to baseline

**Figure 3:**
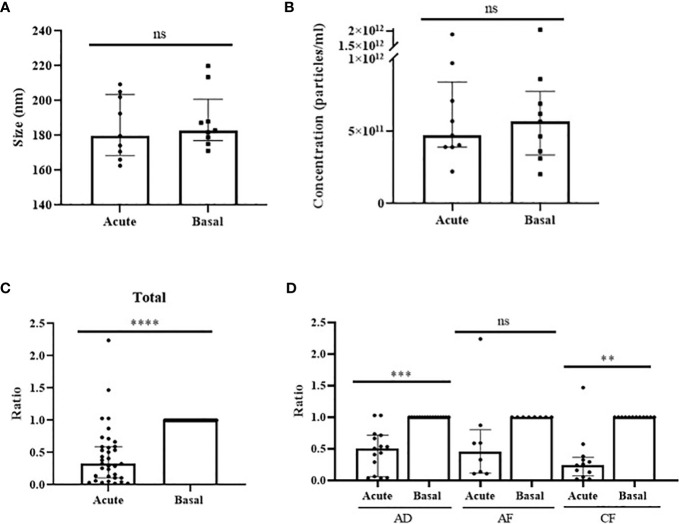
Levels of miR-375-3p within EVs decrease during anaphylaxis. Comparison of particles **(A)** size (*p*=.6523) and **(B)** concentration (*p*>.9999) between EVs obtained from acute and basal phase serum. ns: non-significant. Median ± IQR. **(C)** Levels of miR-375-3p (ratio acute/basal) decrease within EVs during the acute phase of anaphylaxis compared to baseline (****p<.0001; n: 35). Median ± IQR. **(D)** Comparison of changes in vesicular values of miR-375-3p among the three populations studied: adults with drug-induced anaphylaxis (***p=.0003; n: 15), adults with food-induced anaphylaxis (*p*=.1875; n: 8) and children with food-induced anaphylaxis (**p<.0015; n: 12). ns: non-significant. Median ± IQR.

As a great portion of circulating miRNAs is contained in EVs (13), we investigated vesicle-associated miR-375-3p levels in anaphylactic human serum samples. We characterized circulating EVs from acute and baseline stages by different techniques. First, we confirmed vesicles purification by CD9, CD63 and TSG101 immunodetection, all of them considered bona fide protein markers of EVs (**Supplementary Figure 4A**). Transmission em showed heterogeneous size particles consistent previous EVs reports (27) (**Supplementary Figure 4B**). Next, we performed NTA in 9 paired samples (3 adults with drug-induced reactions and 6 (3 adults and 3 children) with food-induced reactions. (**Supplementary Figure 4C**). We did not observe differences in size or concentration when acute and basal vesicles were compared (**Figures 3A, B**).

Next, the hemolysis level of paired samples of 53 patients with anaphylaxis (19 adults with drug-induced reactions and 34 [19 adults and 15 children] with food-induced reactions) was evaluated. The coefficient between miR-23a-3p and miR-451a excluded 23 samples from 18 subjects that were discarded (**Supplementary Figure 4D**). Consequently, the determination of the miRNAs cargo was performed in 35 patients with anaphylaxis (15 adults with drug-induced reactions and 20 [8 adults and 12 children] with food-induced reactions). The correct extraction and reverse transcription of the miRNAs contained in EVs was confirmed using exogenous controls (**Supplementary Figures 4E, F**). Afterwards, the determination of vesicular miR-375-3p levels revealed a decrease during the acute phase of anaphylaxis compared to baseline, similarly as it was observed in serum (**Figure 3C**). Specifically, the independent analysis accordingly to trigger and age showed significant differences in the different cohorts of patients, confirming the drop of this miRNA in the acute phase of anaphylaxis (**Figure 3D**).

### System biology analysis for miR-375-3p and its targets

miR-375-3p serum and EVs-associated reduced levels during anaphylactic reactions could have downstream consequences and participate in the underlying molecular mechanisms of anaphylaxis. Therefore, we conducted an *in silico* SBA on miR-375-3p 269 target genes. The analysis pointed to several *diseases and disorders* related to anaphylaxis pathophysiology, such as gastrointestinal and dermatological diseases. In addition, different *signaling pathways* regulated by miR-375-3p were found to be closely related to the pathological reaction as Phosphoinositide 3-kinases/Protein kinase B (PI3K/Akt), G-Protein coupled receptor (GPCR) and cAMP (**Table 2**).

**Table 2:** *In silico* System Biology Analysis exhibit a possible involvement of miR-375-3p in the molecular mechanisms underlying anaphylaxis.

Diseases and disorders	*p*-Value range
Cancer	1.94e-03 - 3.74e-23
Organismal Injury and Abnormalities	2.15e-03 - 3.74e-23
Gastrointestinal Disease	1.25e-03 - 2.92e-20
Dermatological Diseases and Conditions	1.82e-04 - 5.44e-14
Endocrine System Disorders	1.64e-03 - 1.41e-12
ngenuity canonical pathways	-log(*p*-Value)
IGF-1 Signaling	3.74
Prolactin Signaling	2.53
WNT/β-catenin Signaling	2.44
Protein Ubiquitination Pathway	2.37
STAT3 Pathway	2.35
Molecular Mechanisms of Cancer	2.30
Role of JAK2 in Hormone-like Cytokine Signaling	2.18
PI3K/AKT Signaling	2.11
Role of NANOG in Mammalian Embryonic Stem Cell Pluripotency	1.93
tRNA Splicing	1.87
G-Protein Coupled Receptor Signaling	1.86
Ferroptosis Signaling Pathway	1.84
PEDF Signaling	1.82
cAMP mediated Signaling	1.80
Regulation of eIF4 and p70S6K Signaling	1.78

Top “Diseases and disorders” and the most statistically significant “Ingenuity canonical pathways” obtained from the analysis performed with the IPA software using the 269 miR-375-3p target genes.

**Figure 4:**
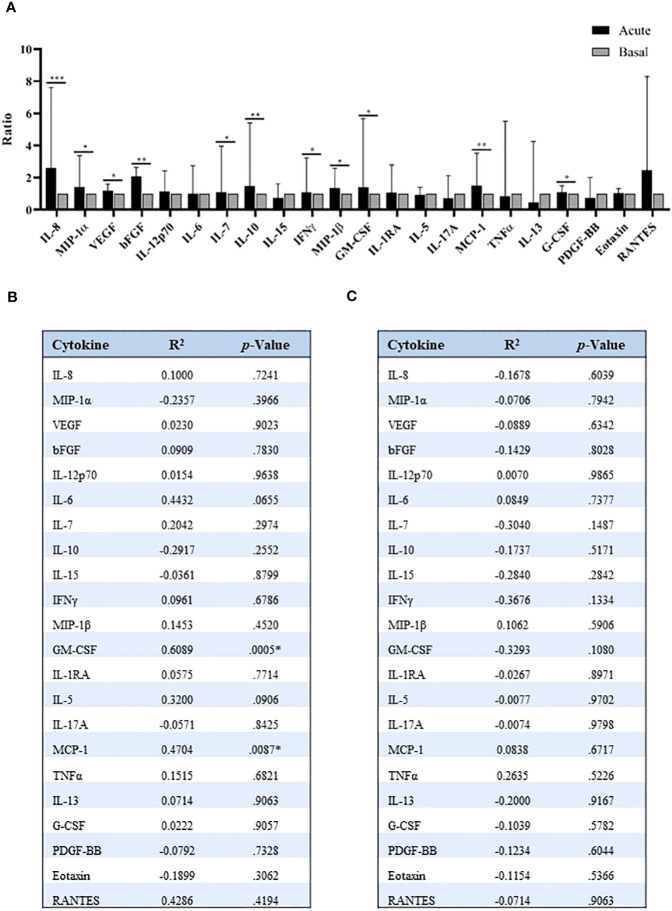
Quantification of serum inflammatory cytokines in patients with anaphylaxis. **(A)** Comparison of acute and basal levels of the 25 inflammatory cytokines identified by multiplex assay: IL-8 (***p=.0007; n: 18), MIP-1α (*p=.0361; n: 19), VEGF (*p=.0322; n: 40), bFGF (**p=.0093; n: 12), IL-12p70 (*p*=.2312; n: 16), IL-6 (*p*=.3270; n: 22), IL-7 (*p=.0394; n: 33), IL-10 (*p=.0290; n: 21), IL-15 (*p*=.9881; n: 23), IFNγ (*p=.0422; n: 26), MIP-1β (*p=.0173; n: 37), GM-CSF (*p=.0105; n: 34), IL-1RA (*p*=.1166; n: 35), IL-5 (*p*=.8620; n: 35), IL-17A (*p*=.3529; n: 19), MCP-1 (**p=.0014; n: 37), TNFα (*p*=.8501; n: 12), IL-13 (*p*=.9453; n: 8), G-CSF (*p=.0159; n: 40), PDGF-BB (*p*=.7317; n: 27), Eotaxin (*p*=.1744; n: 40), RANTES (*p*=.2500; n: 8). IL-1β, IL-2 and IL-4 plots are not shown due to their null detection in most samples. Median ± IQR. Correlation between **(B)** serum and **(C)** vesicular levels of miR-375-3p, and cytokines values. R^2^: Pearson correlation coefficient; bFGF: basic Fibroblast growth factor; G-CSF: granulocyte colony-stimulating factor; GM-CSF: granulocyte macrophage colony-stimulating factor; IFNγ: interferon-γ; IL: interleukin; MCP-1: monocyte chemoattractant protein-1; MIP: macrophage inflammatory protein; PDGF-BB: platelet-derived growth factor-BB; TNFα: tumor necrosis factor-α; VEGF: vascular endothelial growth factor. * Significant difference between groups (p<.05).

Precisely, various of these mechanisms were associated with the inflammatory process, thus we evaluated a panel of inflammatory cytokines to compare their levels with those of miR-375-3p. The analysis performed in serum of patients with anaphylaxis revealed an increase of several of these molecules during the acute phase of the reaction (**Figure 4A**). Furthermore, increased GM-CSF and MCP-1 proteins correlated with decreased serum levels of miR-375-3p, although no association was observed with the other cytokines studied (**Figure 4B**). In turn, no relation was found between miR-375-3p levels within EVs and the molecules assessed by the multiplex assay (**Figure 4C**).

### miR-375-3p partially blocks the cAMP stabilizing effect in the endothelial barrier

**Figure 5:**
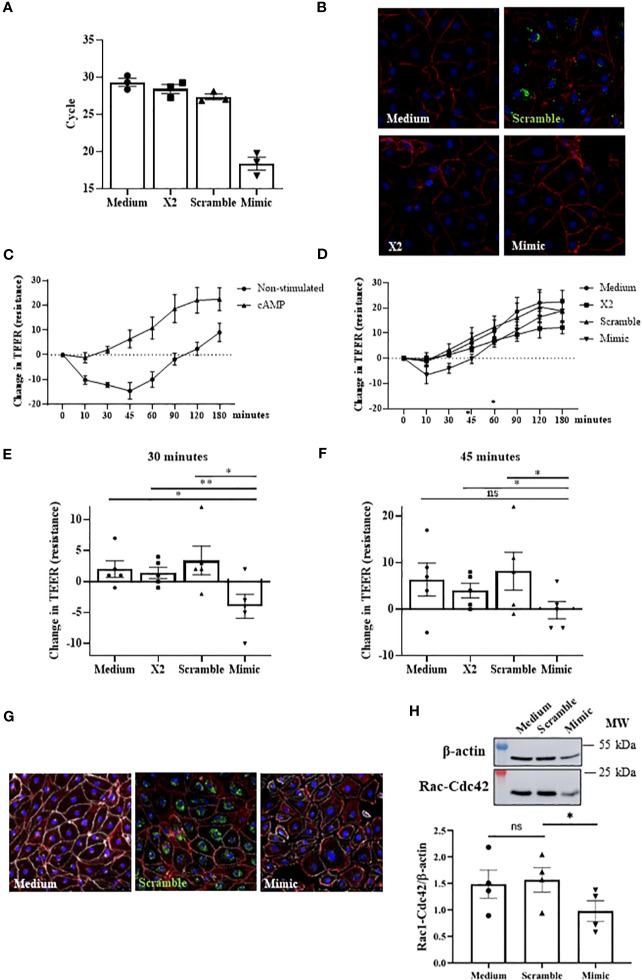
The miR-375-3p partially blocks the stabilizing cAMP response in HMVEC-D. **(A)** Determination of intracellular miRNA levels by qPCR after cells transfection (50mM). **(B)** Immunofluorescence images of transfected HMVEC-D. In red (Texas Red) are visualized the F-actin filaments, in blue (DAPI) the nuclei and in green (fluorescent scramble control) the transfected miRNA. **(C)** Graphic shows the stabilizing effect of cAMP in non-transfected cells (n: 5). **(D)** Changes in TEER measurements in HMVEC-D transfected with miR-375-3p and stimulated with cAMP (n: 5). Significant differences were observed at **(E)** 30 and **(F)** 45 minutes. 30’ miR-375-3p vs medium (*p=.0144), vs X2 (**p=.0090) and vs scramble (*p=.0215); 45’ miR-375-3p vs medium (*p*=.0514), vs X2 (*p=.0167) and vs scramble (*p=.0388). X2: transfection vehicle; ns: non-significant. Mean ± SEM. **(G)** Integrity of endothelial barrier in transfected HMVEC-D evaluated by immunofluorescence performed in 3 independent experiments. Panels show a representative picture of every condition. In red (Texas Red) are visualized the F-actin filaments, in white (Alexa Fluor 647) VE-cadherin, in blue (DAPI) the nuclei and in green (fluorescent scramble control) the transfected miRNA. **(H)** The bottom graphic represents the quantification of Rac1-Cdc42 protein levels in transfected HMVEC-D and the upper panel shows a representative experiment of four performed (*p=.0445).

Increased vascular permeability is a process tightly related to anaphylaxis that involves endothelial barrier rupture. Among the mediators and molecular mechanisms affecting barrier stability, cAMP is crucial for integrity maintenance (11). To evaluate miR-375-3p role in this canonical pathway, we performed *in vitro* endothelial assays. For this purpose, HMVEC-Ds were transfected with an unlabeled mimic to overexpress miR-375-3p. First, the correct performance of the technique was confirmed by measuring the intracellular levels of miR-375-3p and by immunofluorescence images. A high number of miR-375-3p copies was observed by qPCR in those transfected mimic cells (**Figure 5A**), and its corresponding fluorescent labeled scramble was correctly visualized inside cells (**Figure 5B**). Subsequently, we evaluated the functional role of this miRNA on the endothelial barrier integrity. First, the stabilizing effect of cAMP was confirmed by increased values of the cellular monolayer´s resistance in non-transfected ECs (**Figure 5C**). However, cells transfected with miR-375-3p failed to response at the strengthening role exerted by this mediator (**Figure 5D**). Precisely, significant differences in endothelial resistance levels at 30 and 45 minutes were observed in cells transfected with miR-375-3p when compared to control systems (medium, TransIT-X2 and scramble) (**Figures 5E, F**). Next, we tested whether the functional change induced by miR-375-3p was also detectable morphologically via staining of VE-cadherin together with actin filaments (**Figure 5G**). Both in non-transfected ECs and scramble cells, VE-cadherin was localized at cellular membranes and actin was adjacently distributed forming the typical cobblestones cell structure. Contrary, separated cell borders were visualized in miR-375-3p ECs showing VE-cadherin disruption. In addition, miR-375-3p ECs exhibited diminished Rac1-Cdc42 protein levels (**Figure 5H**).

## Discussion

During the last decades, the relevance of miRNAs in allergy has been increasing due to the potential benefits of their use as diagnostic biomarkers, as well as their role regulating a wide variety of molecular processes (33). Accordingly, we have profiled serum circulating miRNAs in drug-mediated anaphylactic reactions and identified decreased serum and EVs-associated levels of miR-375-3p in different groups of patients with anaphylaxis. Furthermore, we have evaluated *in silico* miR-375-3p potential involvement in the reaction and observed a negative role for miR-375-3p in the stability of the endothelial barrier.

Recent studies determined a profile of circulating serum miRNAs in children with food-induced reactions and adults with food- and Hymenoptera venom-mediated anaphylaxis (17, 18). However, no miRNA has been characterized in anaphylactic reactions mediated by drug, even though these compounds are the main elicitors of anaphylaxis in adults (3). Therefore, as NGS offers new possibilities for molecular diagnosis (34), we applied this technology to analyze circulating miRNAs levels in a scarce number of paired serum samples. A total of 21 miRNAs were found statistically different between acute and basal phases, being more than half of them previously described in other allergic diseases. Six of those identified molecules were further studied in a larger and independent cohort of samples from drug mediated anaphylactic reactions using qPCR. The observations showed that only miR-375-3p exhibited statistically significant differences, decreasing in the acute phase of anaphylaxis. Precisely, miR-375-3p reduction has been also described in other allergic diseases such as allergic rhinitis and eosinophilic esophagitis, where it has been proposed as a non-invasive biomarker (35, 36). Additionally, the validation of low levels of miR-375-3p in two extra cohorts of food-mediated anaphylactic reactions patients reinforces its interest. Therefore, this molecule is postulated as a possible molecular marker for the acute phase of anaphylaxis. Moreover, extending the analysis of the miR-375-3p to new samples in future studies would allow the evaluation of its role as a preventive or prognostic biomarker, increasing its clinical relevance. In addition, the use of a set panel including specific proteins, other miRNAs or even metabolites would be a best tool in the clinical practice diagnosing or predicting the risk of reactions.

The systemic nature of anaphylaxis demonstrates the existence of different anaphylactic microenvironments connected through molecular pathways (9). Precisely, secreted miRNAs can mediate communication between different tissues modulating the gene expression of the target cells. In turn, those miRNAs located in EVs are relevant in molecular terms due to the EVs properties as carriers of signaling (13). Therefore, we determined miR-375-3p levels within anaphylactic reaction patient’s serum circulating EVs. The analysis performed demonstrated a significant decrease of this miRNA during the acute phase, as occurred in serum. Respectively, these results demonstrate not only miR-375-3p relevance as a biomarker but also as a molecular player. To our knowledge, this is the first time that miRNA levels have been measured in EVs from patients with anaphylaxis. Therefore, we inquired about EVs-associated miR-375-3p potential role in the underlying mechanisms of the reaction.

Accordingly, we performed SBA to evaluate plausible biological and molecular functions of miR-375-3p in anaphylaxis. The analysis highlighted gastrointestinal and dermatological diseases among the main disorders related to miR-375-3p gene targets. Relevantly, the information obtained associates with anaphylaxis pathophysiology since both systems are altered during this pathological event. Precisely, the skin is the main organ affected during the reaction (80-90%), while the gastrointestinal system is impaired in 45% of cases (37). Canonical pathways analysis exhibited miR-375-3p involvement in relevant molecular mechanisms previously described for anaphylaxis. For instance, this miRNA regulates PI3K/Akt signaling, which is crucial for the degranulation of mast cells and the release of inflammatory mediators (38). Furthermore, this miRNA also modulates Rac signaling, a Ras GTPase that regulates the actin cytoskeleton and controls mast cell degranulation, among other functions (39, 40). Moreover, the miR-375-3p controls Janus kinase 2 (JAK2) signaling which has been linked to atopic dermatitis (41). These receptors are associated with kinases that activate different transcription factors and are involved in both allergic inflammation and Th2 response (42, 43). Precisely, JAK blockade in mast cells prevents immediate hypersensitivity and anaphylactic reactions (44). On the other hand, *in silico* analysis revealed that these miRNAs regulated STAT3 signaling, a transcription factor required for mast cell degranulation (45). In addition, miR-375-3p also modulates GPCRs, a family of receptors activated in anaphylaxis and crucial in the response to vasoactive mediators on the endothelial barrier, modulating vascular permeability and vasodilation (11).

Increased vascular leakage is one of the main alterations underlying anaphylaxis, particularly in severe cases (46). Many vasoactive and proinflammatory molecules are involved in this process causing barrier breakdown (47). EVs purified from anaphylactic patients serum induce an increase in vascular permeability (26). In cancer, miR-375-3p containing EVs are internalized and cause tight junctions (TJs) disruption, altering endothelial barrier integrity through claudin inhibition (48). In addition, other studies in this area of research have shown that miR-375 modulates ECs proliferation, chemotaxis, angiogenesis and inflammation (49, 50). Here, *in-silico* analysis pointed out different miR-375-3p targets pathways that largely converge in the regulation of TJs (51). In particular, the plausible interaction stablished between miR-375 and cAMP signaling attracted our attention because of its central role in the regulation of the endothelial barrier (47). *In vitro*, miR-375-3p transfected cells revealed that its overexpression partially avoids the barrier strengthening induced by cAMP. These monolayers exhibited barrier breakdown accompanied by typical hallmarks such as intercellular gap formation and fragmentation of VE-cadherin staining. Under these conditions, Rac1-Cdc42 protein levels were decreased suggesting the upstream effect of miR-375-3p contributing to the loss of the endothelial barrier properties. In fact, RAC1 target sites has been previously pointed out for miR−375 (52). Therefore, our results show that modulation of miR-375-3p levels and its downstream effects could exert a role in the vascular pathophysiology of anaphylaxis.

Consequently, SBA demonstrated a clear relationship of miR-375-3p with assorted molecular mechanisms related to the pathophysiology of anaphylaxis. However, further studies are needed to confirm the role of this miRNA in each of the signaling pathways studied, as miR-375-3p could have pathological or protective functions. Clarifying these aspects would allow the development of new therapies by increasing or reducing its levels through different pharmacological strategies, such as the use of mimics or sponges, respectively (53).

Anaphylaxis is a systemic reaction where mediators released by effector cells trigger a severe inflammatory scenario (9). In turn, miR-375-3p has been widely implicated in the inflammation subjacent to other pathologies (54). Accordingly, several of the signaling pathways identified in the *in-silico* analysis revealed a possible involvement of this miRNA in the inflammation underlying the anaphylactic reaction. Therefore, we evaluated a panel of inflammatory cytokines to see if their levels correlated with the decrease in miR-375-3p. The results obtained exhibited an increase during the acute phase of several cytokines involved in the inflammatory process, which had been previously described in anaphylaxis (9). In addition, data showed a correlation between the decrease in miR-375-3p levels and the increase in two of the molecules evaluated in the multiplex assay, MCP-1 and GM-CSF. These evidences postulate an association between miR-375-3p and monocytes/macrophages, crucial cells in the alternative pathways of anaphylaxis, especially in IgG-mediated reactions (55, 56). Precisely, miR-375 has been shown to exert an immunoregulatory effect through macrophages (54). Therefore, changes in the levels of this miRNA could be due to the activation of these cells or could be modulating the response of monocytes and macrophages during the anaphylactic reaction.

The recruitment and study of anaphylactic serum samples states several limitations and strengths. First, to mention that the volume of sera harvested in the acute phases was insufficient to use all the patients in each of the approaches carried out in this study. Furthermore, the heterogeneity of the anaphylactic reactions hinders the selection of a representative set of samples for the discovery analysis. However, in this study, 5 well characterized individuals with a specific age range and trigger representing drug anaphylactic reactions, including moderate and severe cases, were chosen for NGS. In the case of miR-375-3p, the results exhibit reproducibility in other cohort of patients with drug-induced anaphylaxis and in adults and children with food anaphylactic reactions. Additionally, the difficulty of obtaining high-quality samples in anaphylaxis hinders obtaining an optimal miRNAs analysis. Red blood cells hemolysis highly influences the release of determined miRNAs (57). Therefore, a strength of our study is the use of abundant controls ensuring to our analysis the elucidation of specific miRNA, free of other cellular contaminants. Specifically, the discarding of hemolyzed samples is essential to avoid unspecific miRNAs detections. In our study, this fact substantially improves the quality of the results, but on the contrary it importantly reduced the number of accurate samples from patients to study. In addition, another limitation is the absence of healthy controls. Accordingly, we consider that it is very complicated to define this group in an acute pathological situation, such as anaphylaxis. Therefore, to overcome this obstacle and to reduce patient heterogeneity, all analyses were performed on a paired manner comparing the acute phase to the baseline phase of the patients, which was used as a control.

In summary, we have identified for the first time a differential profile of circulating serum miRNAs in adults with drug-induced anaphylactic reactions. We have assessed serum and EV-associated miR-375-3p levels in different cohorts of patients, postulating miR-375-3p as a promising molecular marker in anaphylaxis. We have evaluated the possible role of miR-375-3p in the molecular mechanisms of the reaction and studied its functional effect in endothelial barrier stabilization. These results will be validated in larger cohorts to solidify miR-375-3p´s value as a clinical biomarker. In conclusion, and even considering that our findings are exploratory in nature, our results present a clinical potential for the development of new diagnostic and therapeutic strategies.

## Data availability statement

The data presented in the study are deposited in the GEO-NCBI repository, accession number GSE245653.

## Ethics statement

The study was approved by the Ethics Committee (CEIm FJD, PIC057-19), authors adhered to the declaration of Helsinki and all patients were included after giving informed consent by the donors or their parents. The studies were conducted in accordance with the local legislation and institutional requirements. The human samples used in this study were acquired from primarily isolated as part of your previous study for which ethical approval was obtained.

## Author contributions

EN-B, SF-B and AB-M performed the experimental work with participations of LP-G, VDP, AG, MC, LP and JT-A. VE coordinated the work. PRR, ADG, MI-S, JC-H and JJL provided the clinical support and the recruitment of human serum samples. VE, EN-B and ADG designed the experiments and interpreted the results with participations of SF-B and AB-M. VE and EN-B wrote the manuscript. All the authors reviewed and approved the manuscript.

## Glossary

**Table:**

Ad	adults with drug-induced anaphylaxis
Af	adults with food-induced anaphylaxis
cAMP	cyclic AMP
bFGF	basic fibroblast growth factor
cDNA	cyclic DNA
Cdc42	cell division control protein 42 homolog
Cf	children with food-induced anaphylaxis
CNIO	Spanish National Cancer Research Center
DTT	dithiothreitol
ECs	endothelial cells
EM	electron microscopy
EVs	extracellular vesicles
FC	fold change
G-CSF	granulocyte colony-stimulating factor
GAR	goat anti-rabbit
GM-CSF	granulocyte macrophage colony-stimulating factor
GO	gene ontology
GPCR	G-protein coupled receptor
H1R	histamine receptor 1
H2R	histamine receptor 2
HMVEC-D	human dermal microvascular ECs
IFNγ	interferon-γ
IL	interleukin
IPA	Ingenuity Pathway Analysis
IQR	interquartile range
JAK	Janus kinase
MCP-1	monocyte chemoattractant protein-1
MIP-1α	macrophage inflammatory protein-1α
miRNAs	microRNAs
mRNAs	messenger RNAs
NF-κB	nuclear factor kappa-light-chain-enhancer of activated B cells
NGS	next generation sequencing
ns	non-significant
Notch1	neurogenic locus notch homolog protein 1
NTA	nanoparticle tracking analysis
PCA	principal component analysis
PBS	phosphate buffered saline
PDGF-BB	platelet derived growth factor-BB
PFA	paraformaldehyde
PI3K/Akt	phosphoinositide 3-kinases / protein kinase B
PMSF	phenylmethylsulfonyl fluoride
qPCR	quantitative PCR
RAM	rabbit anti-mouse
Rac1	ras-related C3 botulinum toxin substrate 1
RISC	RNA-induced silencing complex
SBA	systems biology analysis
SDS-PAGE	sodium dodecyl sulfate polyacrylamide gels
SEM	standard error of the mean
TE	trisaminomethane buffer
TEER	trans-endothelial electrical resistance
TJ	tight junction
TNFα	tumor necrosis factor α
TWs	Transwell
UMIs	Unique Molecular Identifiers
VEGF	vascular endothelial growth factor
WB	western blot
X2	transfection vehicle
