# Supplementary figures and images for: Novel mediator in anaphylaxis: decreased levels of miR-375-3p in serum and within extracellular vesicles of patients
Source: Front Immunol. 2023 Oct 30;14:1209874. doi: 10.3389/fimmu.2023.1209874 (PMC10642912; doi:10.3389/fimmu.2023.1209874)

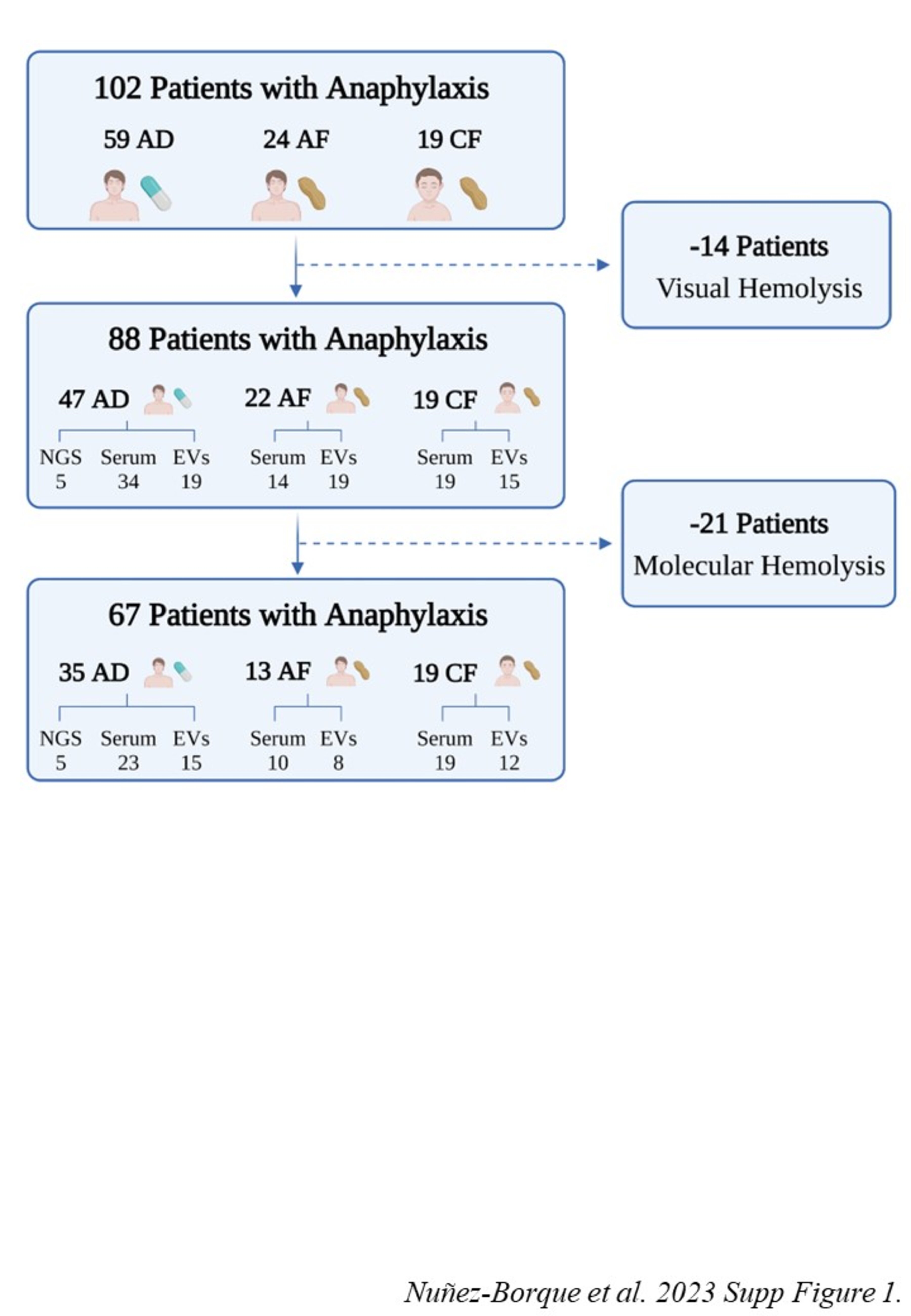

Supplement: Supplementary file 1 [file Image_1.jpeg]

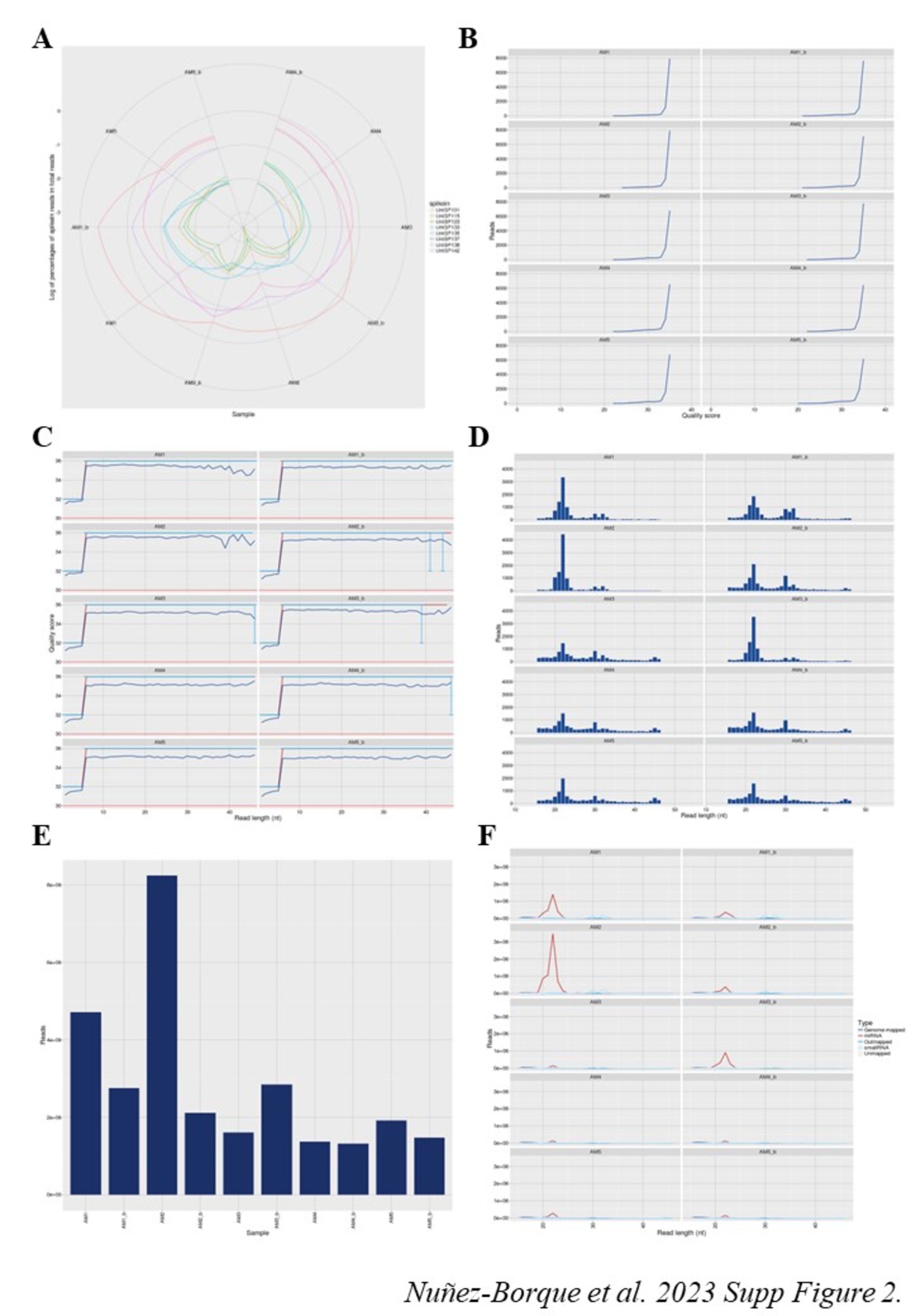

Supplement: Supplementary file 2 [file Image_2.jpeg]

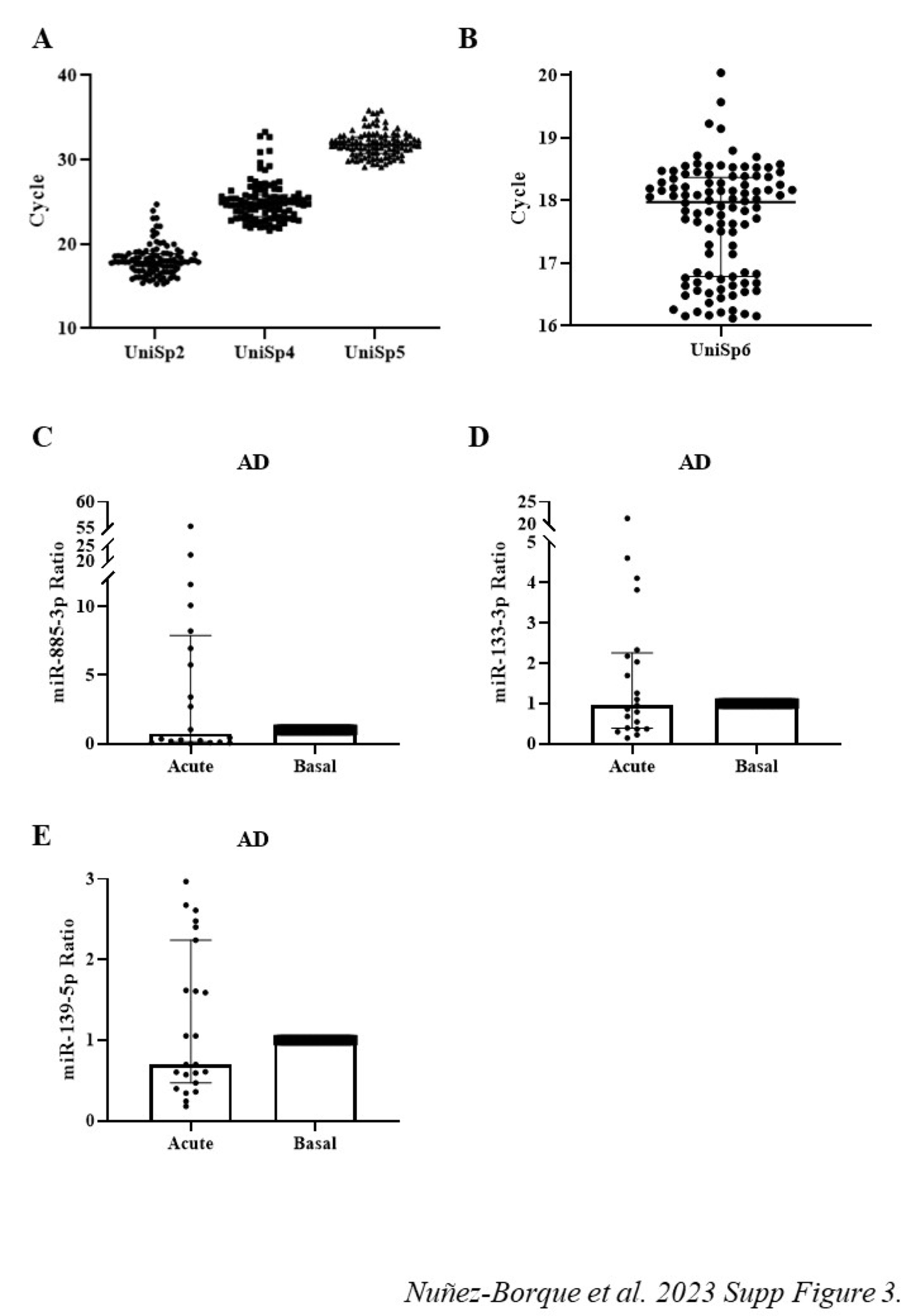

Supplement: Supplementary file 3 [file Image_3.jpeg]

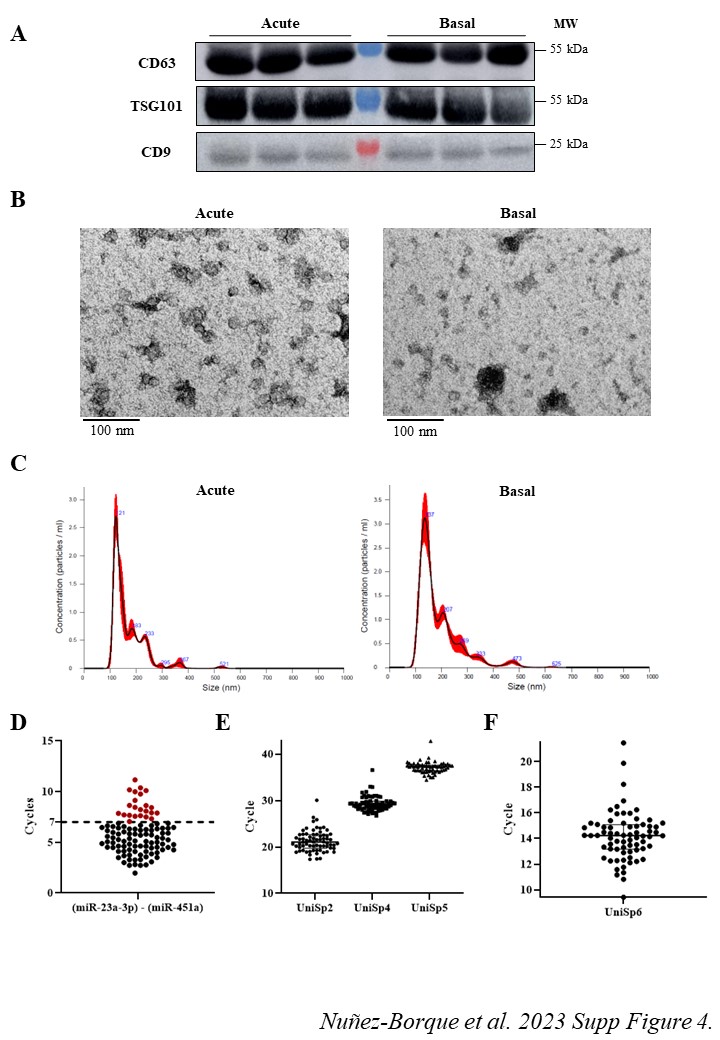

Supplement: Supplementary file 4 [file Image_4.jpeg]
