# Supplementary material for: Novel mediator in anaphylaxis: decreased levels of miR-375-3p in serum and within extracellular vesicles of patients
Source: Front Immunol. 2023 Oct 30;14:1209874. doi: 10.3389/fimmu.2023.1209874 (PMC10642912; doi:10.3389/fimmu.2023.1209874)
Supplement: Supplementary file 5 [file DataSheet_1.docx]

**SUPPLEMENTARY MATERIAL**

**Supplementary Figures legend**

**Supplemental Figure 1.** Flow graph illustrating the exclusion criteria and technical application of the samples. First, 14 patients exhibiting a clearly visible hemolysis in some of their paired samples were ruled out. Next, 21 patients were excluded by molecular hemolysis. In those cases, circulating serum miRNA levels were measured, but exceeded the established threshold for sample quality. Ad: adults with drug-induced anaphylaxis; Af: adults with food-induced anaphylaxis; Cf: children with food-induced anaphylaxis; NGS: next generation sequencing; EVs: extracellular vesicles.

**Supplementary Figure 2.** Quality controls of the NGS. AM: acute phase. AM_b: basal phase. (**A**) Radar plot showing the relative signal of the exogenous controls for the samples. Good correlation of counts between them indicates the correct RNA isolation. (**B**) Average quality of the readings corrected by Unique Molecular Index (UMI). The average Q-score of the readings is plotted on the x-axis and the number of readings on the y-axis. A Q-score greater than 30 (>99.9% correct) is considered high quality data. (**C**) Base quality of the readings corrected by UMI. The x-axis represents the position in the reading, while the y-axis shows the Q-score. The red and the dark blue lines are the median and the mean of the Q-score, respectively. The box plot represents the interquartile range, while the whiskers are the 10% and 90% points. A Q-score greater than 30 is considered high quality data. (**D**) Read length distribution after filtering of the adapters: miRNAs will appear as a peak around 18-23 nucleotides. (**E**) Total number of UMI-corrected reads for each sequenced sample (mean: 2.3 million). (**F**) Read length distribution for each RNA class.

**Supplementary Figure 3.** Exogenous quality controls of the miRNA purification from serum. (**A**) Extraction quality was verified by the exogenous controls UniSp2, UniSp4 and UniSp5. These molecules were detected in a stepwise manner confirming the correct development of the technique (approximately 5-7 cycles). Mean ± SEM. (**B**) Reverse transcription quality was assessed through the exogenous control UniSp6. Mean ± SEM. Comparison (ratio acute/basal) of (**C**) miR-885-3p (*p=*.1429, n: 20), (**D**) miR-133a-3p (*p=*.3377; n: 21) and (**E**) miR-139-5p (*p=*.4961; n: 23) serum levels between acute and basal phases in adults with drug anaphylaxis. The miR-193b-5p and miR-211-5p plots are not shown due to their null detection in most or all samples. ns: non-significant. Median ± IQR.

**Supplementary Figure 4.** Characterization of EVs from patients with drug-induced anaphylaxis and quality controls of the miRNA purification from these particles in all patients included in the study. (**A**) Panels show immunoblots of bona fide EVs markers (CD63, TSG101 and CD9) from 3 representative patients with drug-induced anaphylaxis. MW: molecular weight, KDa: kilodalton. (**B**) Electron microscopy characterization of circulating EVs from both phases of a representative patient with drug-induced anaphylaxis. Scale bar: 100 nm. (**C**) Diagram of the particle size (nm) and concentration (particles/ml) of EVs in a representative adult with drug-induced anaphylaxis. (**D**) Measurement of serum hemolysis by the coefficient between miR-23a-3p and miR-451a in the 106 samples. Among them, 23 exceeded the established threshold (≥7 cycles) and were excluded from the study (red points). (**E**) Extraction quality was verified by stepwise detection (5-7 cycles) of the exogenous controls UniSp2, UniSp4 and UniSp5. Mean ± SEM. (**F**) Reverse transcription quality was assessed through the recognition of the exogenous control UniSp6. Mean ± SEM.

**Supplementary Tables**

| **miRNA** | **log_2_(FC)** | ***p*-Value** | **miRNA** | **log_2_(FC)** | ***p*-Value** | **miRNA** | **log_2_(FC)** | ***p*-Value** |
| --- | --- | --- | --- | --- | --- | --- | --- | --- |
| let-7a-5p | 0.069071 | 0.7603 | miR-196a-5p | -0.469275 | 0.5096 | miR-382-5p | 0.190383 | 0.5178 |
| let-7b-3p | 0.218035 | 0.5714 | miR-196b-5p | 0.133423 | 0.6169 | miR-3940-3p | 0.024350 | 0.8475 |
| let-7b-5p | -0.031017 | 0.8261 | miR-197-3p | -0.270370 | 0.5035 | miR-409-3p | 0.034062 | 0.8135 |
| let-7c-5p | -0.190575 | 0.5943 | miR-1976 | -0.329829 | 0.4114 | miR-411-5p | 0.705820 | 0.0730 |
| let-7d-3p | 0.072006 | 0.7249 | miR-199a-3p | -0.030411 | 0.8269 | miR-421 | -0.190544 | 0.6371 |
| let-7d-5p | 0.252070 | 0.4664 | miR-199a-5p | -0.154820 | 0.6725 | miR-423-3p | 0.214486 | 0.4690 |
| let-7e-5p | -0.035692 | 0.8148 | miR-199b-3p | 0.241789 | 0.5016 | miR-423-5p | -0.159787 | 0.5963 |
| let-7f-5p | 0.028657 | 0.8282 | miR-199b-5p | -0.095563 | 0.7568 | miR-424-5p | -0.736364 | 0.1019 |
| let-7g-5p | 0.071606 | 0.7506 | miR-19a-3p | 0.073962 | 0.7290 | miR-425-3p | 0.003144 | 0.8715 |
| let-7i-3p | 0.290376 | 0.4944 | miR-19b-3p | 0.002563 | 0.8723 | miR-425-5p | -0.054685 | 0.7799 |
| let-7i-5p | -0.066703 | 0.7469 | miR-200a-3p | -0.996413 | 0.1321 | miR-431-5p | 0.041325 | 0.8321 |
| miR-1 | -0.738591 | 0.1438 | miR-200b-3p | -0.167969 | 0.6745 | miR-432-5p | -0.051718 | 0.7834 |
| miR-100-5p | -0.718176 | 0.2238 | miR-200c-3p | -0.194600 | 0.6063 | miR-4433b-3p | 1.352501 | 0.0668 |
| miR-101-3p | -0.217631 | 0.5126 | miR-203a | -1.282744 | 0.0489 | miR-4433b-5p | -0.063181 | 0.8000 |
| miR-103a-3p | 0.575460 | 0.1278 | miR-204-5p | -0.810242 | 0.1451 | miR-4446-3p | 2.114556 | 0.0021 |
| miR-106a-5p | -0.475697 | 0.3043 | miR-205-5p | -1.187336 | 0.0079 | miR-451a | 0.186560 | 0.6693 |
| miR-106b-3p | -0.112800 | 0.7349 | miR-206 | -1.240342 | 0.0106 | miR-454-3p | 0.543781 | 0.1269 |
| miR-106b-5p | 0.397128 | 0.4350 | miR-20a-5p | 0.037065 | 0.8074 | miR-454-5p | -0.011961 | 0.8586 |
| miR-107 | 0.283652 | 0.4269 | miR-20b-5p | 0.578373 | 0.1625 | miR-4685-3p | -2.189190 | 0.0008 |
| miR-10a-5p | -0.420468 | 0.2080 | miR-210-3p | 0.431875 | 0.3749 | miR-4732-3p | 0.173515 | 0.6371 |
| miR-10b-3p | 0.596225 | 0.5478 | miR-2110 | -0.452439 | 0.2107 | miR-4732-5p | 0.192755 | 0.5769 |
| miR-10b-5p | -0.537496 | 0.2006 | miR-211-5p | 2.386044 | 0.0002 | miR-4742-3p | -0.481275 | 0.4739 |
| miR-1180-3p | 0.102355 | 0.7209 | miR-2116-3p | 0.447504 | 0.4393 | miR-483-3p | -1.100157 | 0.1344 |
| miR-1224-5p | 0.183222 | 0.6648 | miR-215-5p | -1.073399 | 0.0516 | miR-483-5p | -1.224310 | 0.0643 |
| miR-122-5p | -1.376949 | 0.0439 | miR-21-5p | -0.115423 | 0.6587 | miR-484 | -0.409824 | 0.3661 |
| miR-1226-3p | -0.860130 | 0.0496 | miR-221-3p | 0.173677 | 0.5098 | miR-485-3p | -0.346491 | 0.3747 |
| miR-1247-5p | -1.809141 | 0.0047 | miR-221-5p | 1.087699 | 0.0913 | miR-485-5p | 0.641125 | 0.2552 |
| miR-1249 | -0.926631 | 0.1972 | miR-222-3p | 0.185097 | 0.5845 | miR-486-3p | -0.209113 | 0.5839 |
| miR-1255b-5p | 0.094417 | 0.7521 | miR-223-3p | -0.076113 | 0.7788 | miR-486-5p | -0.028961 | 0.8284 |
| miR-125a-3p | 0.411029 | 0.4925 | miR-223-5p | 0.083680 | 0.7615 | miR-487b-3p | 0.591404 | 0.1335 |
| miR-125a-5p | -0.686787 | 0.1150 | miR-22-3p | 0.051628 | 0.7955 | miR-491-5p | -0.357980 | 0.4413 |
| miR-125b-5p | -0.766131 | 0.0906 | miR-224-5p | 0.989984 | 0.0941 | miR-493-3p | 0.674432 | 0.0627 |
| miR-1260a | -0.329748 | 0.5020 | miR-23a-3p | -0.079702 | 0.7388 | miR-493-5p | -0.296222 | 0.6365 |
| miR-1260b | -0.388808 | 0.4333 | miR-23a-5p | 0.093212 | 0.7973 | miR-494-3p | 0.487107 | 0.3606 |
| miR-126-3p | 0.163855 | 0.5868 | miR-23b-3p | -0.061381 | 0.7717 | miR-497-5p | -0.639585 | 0.3850 |
| miR-126-5p | 0.033085 | 0.8266 | miR-23b-5p | -0.450979 | 0.3369 | miR-500a-3p | 0.005955 | 0.8696 |
| miR-1270 | -0.372052 | 0.4245 | miR-24-3p | 0.007043 | 0.8666 | miR-5010-5p | 0.176068 | 0.6396 |
| miR-1273h-3p | 0.059674 | 0.7945 | miR-25-3p | 0.081160 | 0.7696 | miR-501-3p | -0.111687 | 0.6878 |
| miR-127-3p | -0.452242 | 0.3426 | miR-25-5p | -0.691264 | 0.1151 | miR-502-3p | 0.048791 | 0.8331 |
| miR-1277-5p | 1.009299 | 0.0933 | miR-26a-1-3p | 0.761618 | 0.1794 | miR-503-5p | 0.504765 | 0.3583 |
| miR-128-3p | 0.138896 | 0.6352 | miR-26a-5p | 0.126765 | 0.6516 | miR-504-5p | -1.481258 | 0.1716 |
| miR-1287-5p | 0.312025 | 0.5365 | miR-26b-3p | 0.418366 | 0.3386 | miR-505-3p | -0.601395 | 0.2636 |
| miR-1292-5p | 0.765180 | 0.2899 | miR-26b-5p | 0.134560 | 0.6268 | miR-505-5p | -0.010162 | 0.8605 |
| miR-1294 | 0.176928 | 0.6374 | miR-27a-3p | 0.150394 | 0.6364 | miR-5187-5p | 0.756120 | 0.1394 |
| miR-1296-5p | 0.035296 | 0.8182 | miR-27b-3p | -0.304097 | 0.3387 | miR-5189-3p | 0.557537 | 0.0690 |
| miR-1299 | -1.681144 | 0.1782 | miR-28-3p | 0.145788 | 0.6373 | miR-532-3p | 0.096647 | 0.6878 |
| miR-1301-3p | 0.667758 | 0.1961 | miR-296-5p | 0.277969 | 0.5443 | miR-532-5p | -0.003355 | 0.8715 |
| miR-1304-3p | -0.147764 | 0.6732 | miR-29a-3p | -0.300193 | 0.3555 | miR-542-3p | -0.607648 | 0.2207 |
| miR-1306-5p | -0.770848 | 0.1842 | miR-29b-3p | 0.076254 | 0.7522 | miR-542-5p | 0.532970 | 0.5735 |
| miR-1307-3p | -0.248587 | 0.4179 | miR-29c-3p | -0.063067 | 0.7800 | miR-548j-5p | 0.625740 | 0.2974 |
| miR-1307-5p | 0.219667 | 0.5952 | miR-29c-5p | -0.005299 | 0.8706 | miR-550a-3p | -0.145654 | 0.7702 |
| miR-130a-3p | 0.417549 | 0.3125 | miR-301a-3p | 0.931053 | 0.0911 | miR-574-3p | -0.113145 | 0.6774 |
| miR-130b-3p | -0.033665 | 0.8246 | miR-3065-5p | -0.071506 | 0.7320 | miR-576-5p | 0.025346 | 0.8425 |
| miR-130b-5p | 1.027914 | 0.1284 | miR-30a-3p | -0.767051 | 0.0969 | miR-584-5p | 0.509233 | 0.2123 |
| miR-132-3p | 0.252045 | 0.5445 | miR-30a-5p | -0.459351 | 0.2394 | miR-589-5p | 0.001610 | 0.8737 |
| miR-133a-3p | 1.495741 | 0.0246 | miR-30b-5p | 0.351369 | 0.4366 | miR-590-3p | -0.323916 | 0.4870 |
| miR-134-5p | 0.180677 | 0.5775 | miR-30c-5p | 0.530553 | 0.1693 | miR-598-3p | 0.891331 | 0.0693 |
| miR-139-3p | 0.076641 | 0.7878 | miR-30d-5p | -0.020210 | 0.8365 | miR-625-3p | 0.327878 | 0.3027 |
| miR-139-5p | 1.525966 | 0.0069 | miR-30e-3p | 0.471340 | 0.2195 | miR-625-5p | 0.228889 | 0.5106 |
| miR-140-3p | 0.175207 | 0.6523 | miR-30e-5p | -0.040814 | 0.7993 | miR-627-5p | 0.953823 | 0.1888 |
| miR-140-5p | 0.110664 | 0.7291 | miR-3138 | 0.262638 | 0.4534 | miR-628-3p | -0.226222 | 0.5412 |
| miR-141-3p | -0.864631 | 0.1958 | miR-3150b-3p | -1.978972 | 0.0009 | miR-629-5p | 0.364777 | 0.3043 |
| miR-142-3p | -0.194755 | 0.6012 | miR-3158-3p | 0.607263 | 0.2945 | miR-636 | -0.937222 | 0.1351 |
| miR-142-5p | 0.030943 | 0.8242 | miR-31-5p | -0.541684 | 0.6104 | miR-642a-3p | 0.231988 | 0.5489 |
| miR-143-3p | -0.084781 | 0.7531 | miR-3173-5p | -1.235327 | 0.0246 | miR-642a-5p | 0.561189 | 0.5750 |
| miR-143-5p | -0.739157 | 0.2810 | miR-3187-3p | 0.121481 | 0.7026 | miR-6511a-3p | -0.140498 | 0.8054 |
| miR-144-3p | 0.206967 | 0.5222 | miR-3200-3p | 0.722790 | 0.4423 | miR-6511b-3p | 0.123235 | 0.7557 |
| miR-144-5p | 0.456150 | 0.2684 | miR-320a | -0.012861 | 0.8531 | miR-6513-3p | 2.178829 | 0.0020 |
| miR-145-5p | 0.160529 | 0.6590 | miR-320b | -0.656243 | 0.0616 | miR-652-3p | 0.228096 | 0.5334 |
| miR-1468-5p | -0.496227 | 0.3959 | miR-320c | -0.692849 | 0.0447 | miR-654-3p | 0.151467 | 0.6969 |
| miR-146a-5p | 0.442461 | 0.2529 | miR-320d | -0.703180 | 0.1187 | miR-660-5p | -0.166153 | 0.5983 |
| miR-146b-3p | 0.175384 | 0.5830 | miR-323a-3p | -0.212629 | 0.7166 | miR-664a-3p | 1.155560 | 0.0567 |
| miR-146b-5p | 0.130550 | 0.6683 | miR-323b-3p | 0.573948 | 0.4737 | miR-664a-5p | 0.352217 | 0.3335 |
| miR-148a-3p | -0.450634 | 0.2099 | miR-324-3p | 0.436661 | 0.1897 | miR-664b-3p | -0.429831 | 0.3965 |
| miR-148b-3p | 0.129291 | 0.6595 | miR-324-5p | 0.313405 | 0.4356 | miR-664b-5p | 0.915437 | 0.0572 |
| miR-148b-5p | 0.126639 | 0.6604 | miR-32-5p | 0.008609 | 0.8617 | miR-671-3p | 0.567679 | 0.1995 |
| miR-150-3p | -0.265968 | 0.5611 | miR-326 | 1.363800 | 0.0354 | miR-671-5p | -0.509844 | 0.2241 |
| miR-150-5p | -0.541543 | 0.1843 | miR-328-3p | -0.206509 | 0.6265 | miR-6721-5p | 1.048590 | 0.2183 |
| miR-151a-3p | 0.558763 | 0.1139 | miR-330-3p | 0.170503 | 0.7017 | miR-6734-5p | 0.379225 | 0.4168 |
| miR-151a-5p | 0.428688 | 0.3764 | miR-331-3p | 0.858900 | 0.0881 | miR-6780a-5p | -1.298282 | 0.0972 |
| miR-152-3p | 0.030023 | 0.8230 | miR-335-3p | 0.379977 | 0.3698 | miR-6786-3p | 0.051315 | 0.8088 |
| miR-155-5p | 0.051675 | 0.7967 | miR-335-5p | 0.370412 | 0.3872 | miR-6803-3p | 0.582003 | 0.2165 |
| miR-15a-5p | 0.253263 | 0.4953 | miR-338-3p | 0.376716 | 0.4116 | miR-6842-3p | 0.279435 | 0.5476 |
| miR-15b-3p | 0.688222 | 0.2566 | miR-339-3p | 0.528016 | 0.1815 | miR-6852-5p | 1.527983 | 0.0183 |
| miR-15b-5p | 0.034329 | 0.8260 | miR-339-5p | 0.197496 | 0.5432 | miR-6884-5p | -0.314967 | 1.0000 |
| miR-16-2-3p | -0.288052 | 0.5781 | miR-340-5p | 0.296875 | 0.4800 | miR-744-5p | 0.104219 | 0.7125 |
| miR-16-5p | 0.342485 | 0.2942 | miR-342-3p | -0.306638 | 0.3926 | miR-7-5p | 0.054504 | 0.7793 |
| miR-17-5p | 0.299901 | 0.3848 | miR-342-5p | -0.627038 | 0.1569 | miR-760 | 0.592212 | 0.2036 |
| miR-181a-2-3p | 0.128332 | 0.6474 | miR-345-5p | -0.012541 | 0.8555 | miR-766-3p | 0.704260 | 0.1241 |
| miR-181a-3p | 0.246936 | 0.6271 | miR-34a-5p | -0.646707 | 0.1614 | miR-766-5p | 0.287398 | 0.6292 |
| miR-181a-5p | 0.178079 | 0.5615 | miR-3605-3p | -0.151099 | 0.6014 | miR-769-5p | 0.110407 | 0.7183 |
| miR-181b-5p | 0.103452 | 0.7058 | miR-3613-5p | -0.320761 | 0.3381 | miR-7706 | 0.423977 | 0.3302 |
| miR-181c-3p | -0.197255 | 0.6139 | miR-361-3p | 0.145746 | 0.6565 | miR-7976 | -0.173352 | 0.7420 |
| miR-181d-5p | -0.549811 | 0.4643 | miR-3615 | -0.496944 | 0.2380 | miR-874-3p | -0.314691 | 0.4350 |
| miR-182-5p | 0.446666 | 0.2986 | miR-361-5p | -0.084216 | 0.7316 | miR-877-5p | -0.775968 | 0.3243 |
| miR-183-5p | 0.252651 | 0.5477 | miR-362-5p | 1.382777 | 1.0000 | miR-885-3p | -2.573851 | 0.0041 |
| miR-184 | -1.137839 | 0.0578 | miR-363-3p | 0.257660 | 0.5424 | miR-885-5p | -1.203418 | 0.0322 |
| miR-185-3p | 0.570785 | 0.2483 | miR-365a-3p | -0.615436 | 0.2521 | miR-92a-3p | -0.085079 | 0.7460 |
| miR-185-5p | 0.251592 | 0.4904 | miR-365b-3p | -0.474534 | 0.2276 | miR-92b-3p | -0.438234 | 0.4222 |
| miR-186-5p | 0.440239 | 0.2615 | miR-3679-5p | 0.211360 | 0.7076 | miR-92b-5p | -0.155304 | 0.6354 |
| miR-18a-3p | -0.035746 | 0.8301 | miR-3688-3p | -0.278715 | 0.6502 | miR-93-3p | -0.034776 | 0.8336 |
| miR-18a-5p | 0.663302 | 0.1370 | miR-369-3p | 0.134898 | 0.6953 | miR-93-5p | 0.301314 | 0.4138 |
| miR-1908-5p | 0.112843 | 0.7253 | miR-369-5p | 0.197415 | 0.6335 | miR-941 | 0.473328 | 0.2679 |
| miR-190a-5p | 0.647278 | 0.1281 | miR-370-3p | -0.365836 | 0.3348 | miR-942-5p | -0.164491 | 0.6789 |
| miR-190b | 0.129435 | 0.7354 | miR-374a-3p | -0.781314 | 0.3490 | miR-95-3p | -0.522917 | 0.3532 |
| miR-191-3p | -0.108380 | 0.7352 | miR-374a-5p | 0.363112 | 0.3602 | miR-96-5p | -0.220507 | 0.6530 |
| miR-191-5p | 0.380631 | 0.3655 | miR-374b-5p | 1.019815 | 0.0772 | miR-98-5p | 0.303162 | 0.3754 |
| miR-192-5p | -0.333480 | 0.3492 | miR-375-3p | -1.631319 | 0.0034 | miR-99a-5p | -0.624709 | 0.1067 |
| miR-193a-5p | -0.731579 | 0.1551 | miR-378a-3p | -0.299834 | 0.4288 | miR-99b-3p | -0.077283 | 0.7452 |
| miR-193b-5p | -1.895626 | 0.0072 | miR-378a-5p | -0.603173 | 0.2644 | miR-99b-5p | -0.087804 | 0.7088 |
| miR-194-5p | -0.282132 | 0.3727 | miR-379-5p | 0.482040 | 0.2603 |  |  |  |
| miR-195-5p | -0.242912 | 0.5163 | miR-381-3p | 0.348292 | 0.3254 |  |  |  |

**Supplementary Table 1.** List of all circulating miRNAs identified by NGS in serum from adults with drug anaphylaxis. FC: fold change (acute/basal phase). Log_2_(FC): positive values indicate an increase (red) while negative values imply a decrease (blue) in the acute phase.

| **miRNA** | **Sequence** | **log_2_(FC)** | ***p*-Value** | **FDR** |
| --- | --- | --- | --- | --- |
| miR-211-5p | UUCCCUUUGUCAUCCUUCGCCU | 2.38604 | 0.00023 | 0.00000 |
| miR-6513-3p | UCAAGUGUCAUCUGUCCCUAG | 2.17883 | 0.00198 | 0.00000 |
| miR-4446-3p | CAGGGCUGGCAGUGACAUGGGU | 2.11456 | 0.00211 | 0.00000 |
| miR-6852-5p | CCCUGGGGUUCUGAGGACAUG | 1.52798 | 0.01834 | 0.30769 |
| miR-139-5p | UCUACAGUGCACGUGUCUCCAGU | 1.52597 | 0.00688 | 0.09091 |
| miR-133a-3p | UUUGGUCCCCUUCAACCAGCUG | 1.49574 | 0.02456 | 0.40000 |
| miR-326 | CCUCUGGGCCCUUCCUCCAG | 1.36380 | 0.03544 | 0.52941 |
| miR-320c | AAAAGCUGGGUUGAGAGGGU | -0.69285 | 0.04470 | 0.57895 |
| miR-1226-3p | UCACCAGCCCUGUGUUCCCUAG | -0.86013 | 0.04962 | 0.61290 |
| miR-205-5p | UCCUUCAUUCCACCGGAGUCUG | -1.18734 | 0.00788 | 0.09091 |
| miR-885-5p | UCCAUUACACUACCCUGCCUCU | -1.20342 | 0.03221 | 0.50000 |
| miR-3173-5p | UGCCCUGCCUGUUUUCUCCUUU | -1.23533 | 0.02463 | 0.40000 |
| miR-206 | UGGAAUGUAAGGAAGUGUGUGG | -1.24034 | 0.01065 | 0.16667 |
| miR-203a | GUGAAAUGUUUAGGACCACUAG | -1.28274 | 0.04891 | 0.61290 |
| miR-122-5p | UGGAGUGUGACAAUGGUGUUUG | -1.37695 | 0.04389 | 0.57895 |
| miR-375-3p | UUUGUUCGUUCGGCUCGCGUGA | -1.63132 | 0.00337 | 0.00000 |
| miR-1247-5p | ACCCGUCCCGUUCGUCCCCGGA | -1.80914 | 0.00468 | 0.09091 |
| miR-193b-5p | CGGGGUUUUGAGGGCGAGAUGA | -1.89563 | 0.00723 | 0.09091 |
| miR-3150b-3p | UGAGGAGAUCGUCGAGGUUGG | -1.97897 | 0.00091 | 0.00000 |
| miR-4685-3p | UCUCCCUUCCUGCCCUGGCUAG | -2.18919 | 0.00079 | 0.00000 |
| miR-885-3p | AGGCAGCGGGGUGUAGUGGAUA | -2.57385 | 0.00413 | 0.09091 |

**Supplementary Table 2.** List of the 21 statistically significant miRNAs between the acute and basal phase of adults with drug anaphylaxis. FC: fold change (acute/basal phase). Log_2_(FC): positive values indicate an increase (red) while negative values imply a decrease (blue) in the acute phase. FDR: False Discovery Rate.

| **miRNA** | **Allergic conditions** |
| --- | --- |
| miR-211-5p | As (↓) (1) |
| miR-6513-3p | - |
| miR-4446-3p | - |
| miR-6852-5p | - |
| miR-139-5p | As, (↓↑) AD (↑) (2–6) |
| miR-133a-3p | As (↓↑) (7–11) |
| miR-326 | As (↓) (12) |
| miR-320c | As (↓) (13,14) |
| miR-1226-3p | - |
| miR-205-5p | AR (↑), As (↑) (15–17) |
| miR-885-5p | - |
| miR-3173-5p | - |
| miR-206 | As (↓↑ ), AR (↑) (18–27) |
| miR-203a | As (↓↑ ) (27–31) |
| miR-122-5p | As (↑) (32) |
| miR-375 | As (↓), AD (↓↑ ), EoE (↓), AR (↓↑) (33–42) |
| miR-1247-5p | - |
| miR-193b-5p | As (↓), EoE (↓), AD (↓) (38,43) |
| miR-3150b-3p | - |
| miR-4685-3p | - |
| miR-885-3p | As (↓) (44) |

**Supplementary Table 3.** Background in the field of allergy of the 21 significant miRNAs identified by NGS. The entire search has been performed in Pubmed. As: asthma; AD: atopic dermatitis; EoE: eosinophilic esophagitis; AR: allergic rhinitis. ↑ Increased miRNA. ↓ Decreased miRNA.

**Supplementary References**

1. Zeng H, Gao H, Zhang M, Wang J, Gu Y, Wang Y et al. Atractylon Treatment Attenuates Pulmonary Fibrosis via Regulation of the mmu_circ_0000981/miR-211-5p/TGFBR2 Axis in an Ovalbumin-Induced Asthma Mouse Model. *Inflammation* 2021;**44**:1856–1864.

2. Zhu H, Wang K, Yang L, Xu Q, Ren F, Liu X. [Yanghe Pingchuan granule promotes BMSCs homing in asthmatic rats by upregulating miR-139-5p and downregulating Notch1/Hes1 pathway]. *Nan Fang Yi Ke Da Xue Xue Bao* 2020;**40**:1703–1711.

3. Zhang H, Sun Z, Yu L, Sun J. MiR-139-5p inhibits proliferation and promoted apoptosis of human airway smooth muscle cells by downregulating the Brg1 gene. *Respir Physiol Neurobiol* 2017;**246**:9–16.

4. Quan L, Ren G, Liu L, Huang W, Li M. Circular RNA circ_0002594 regulates PDGF-BB-induced proliferation and migration of human airway smooth muscle cells via sponging miR-139-5p/TRIM8 in asthma. *Autoimmunity* 2022;**55**:339–350.

5. Bao L, Chau CS, Lei Z, Hu H, Chan AG, Amber KT et al. Dysregulated microRNA expression in IL-4 transgenic mice, an animal model of atopic dermatitis. *Arch Dermatol Res* 2021;**313**:837–846.

6. Kho AT, Sharma S, Davis JS, Spina J, Howard D, McEnroy K et al. Circulating MicroRNAs: Association with Lung Function in Asthma. *PloS One* 2016;**11**:e0157998.

7. Chen Y, Mao Z-D, Shi Y-J, Qian Y, Liu Z-G, Yin X-W et al. Comprehensive analysis of miRNA-mRNA-lncRNA networks in severe asthma. *Epigenomics* 2019;**11**:115–131.

8. Mendes FC, Paciência I, Ferreira AC, Martins C, Rufo JC, Silva D et al. Development and validation of exhaled breath condensate microRNAs to identify and endotype asthma in children. *PloS One* 2019;**14**:e0224983.

9. Mendes FC, Paciência I, Cavaleiro Rufo J, Silva D, Delgado L, Moreira A et al. Dietary Acid Load Modulation of Asthma-Related miRNAs in the Exhaled Breath Condensate of Children. *Nutrients* 2022;**14**:1147.

10. Pinkerton M, Chinchilli V, Banta E, Craig T, August A, Bascom R et al. Differential expression of microRNAs in exhaled breath condensates of patients with asthma, patients with chronic obstructive pulmonary disease, and healthy adults. *J Allergy Clin Immunol* 2013;**132**:217–219.

11. Shao Y, Chong L, Lin P, Li H, Zhu L, Wu Q et al. MicroRNA-133a alleviates airway remodeling in asthtama through PI3K/AKT/mTOR signaling pathway by targeting IGF1R. *J Cell Physiol* 2019;**234**:4068–4080.

12. Lin J, Feng X, Zhang J. Circular RNA circHIPK3 modulates the proliferation of airway smooth muscle cells by miR-326/STIM1 axis. *Life Sci* 2020;**255**:117835.

13. Bersimbaev R, Aripova A, Bulgakova O, Kussainova А, Akparova A, Izzotti A. The Plasma Levels of hsa-miR-19b-3p, hsa-miR-125b-5p, and hsamiR- 320c in Patients with Asthma, COPD and Asthma-COPD Overlap Syndrome (ACOS). *MicroRNA Shariqah United Arab Emir* 2021;**10**:130–138.

14. Aripova A, Akparova A, Bersimbaev R. The Potential Role of miRNA-19b-3p and miRNA-320c in Patients with Moderate Bronchial Asthma. *MicroRNA Shariqah United Arab Emir* 2020;**9**:373–377.

15. Zhang S, Lin S, Tang Q, Yan Z. Knockdown of miR‑205‑5p alleviates the inflammatory response in allergic rhinitis by targeting B‑cell lymphoma 6. *Mol Med Rep* 2021;**24**:818.

16. Kuang Y, Hu B, Huang M, Zhao S, Wu X, Zhang M et al. Phosphatidylethanolamine-binding protein 1 (PEBP1) mediates the regulatory role of microRNAs (miRNAs)-205-5p in degranulation and histamine release. *Bioengineered* 2022;**13**:13341–13351.

17. Suojalehto H, Toskala E, Kilpeläinen M, Majuri M-L, Mitts C, Lindström I et al. MicroRNA profiles in nasal mucosa of patients with allergic and nonallergic rhinitis and asthma. *Int Forum Allergy Rhinol* 2013;**3**:612–620.

18. Zhang K, Feng Y, Liang Y, Wu W, Chang C, Chen D et al. Epithelial miR-206 targets CD39/extracellular ATP to upregulate airway IL-25 and TSLP in type 2-high asthma. *JCI Insight* 2021;**6**:148103.

19. Lin C-C, Law BF, Hettick JM. Acute 4,4’-Methylene Diphenyl Diisocyanate Exposure-Mediated Downregulation of miR-206-3p and miR-381-3p Activates Inducible Nitric Oxide Synthase Transcription by Targeting Calcineurin/NFAT Signaling in Macrophages. *Toxicol Sci Off J Soc Toxicol* 2020;**173**:100–113.

20. Yin H, Liu MH, Gao F, Shang HM. Pro-inflammatory and pro-fibrotic role of long non-coding RNA RMRP in pediatric asthma through targeting microRNA-206/CCL2 axis. *J Biol Regul Homeost Agents* 2021;**35**:71–83.

21. Wang L, Xu J, Liu H, Li J, Hao H. PM2.5 inhibits SOD1 expression by up-regulating microRNA-206 and promotes ROS accumulation and disease progression in asthmatic mice. *Int Immunopharmacol* 2019;**76**:105871.

22. Kho AT, McGeachie MJ, Moore KG, Sylvia JM, Weiss ST, Tantisira KG. Circulating microRNAs and prediction of asthma exacerbation in childhood asthma. *Respir Res* 2018;**19**:128.

23. Chen R, Piao L-Z, Liu L, Zhang X-F. DNA methylation and gene expression profiles to identify childhood atopic asthma associated genes. *BMC Pulm Med* 2021;**21**:292.

24. Tian J, Ning J, Xu Y. [Bioinformatics analysis of differentially expressed microRNAs in children with bronchial asthma]. *Xi Bao Yu Fen Zi Mian Yi Xue Za Zhi Chin J Cell Mol Immunol* 2021;**37**:923–931.

25. Panganiban RP, Wang Y, Howrylak J, Chinchilli VM, Craig TJ, August A et al. Circulating microRNAs as biomarkers in patients with allergic rhinitis and asthma. *J Allergy Clin Immunol* 2016;**137**:1423–1432.

26. Lin C-C, Law BF, Hettick JM. MicroRNA-mediated calcineurin signaling activation induces CCL2, CCL3, CCL5, IL8, and chemotactic activities in 4,4’-methylene diphenyl diisocyanate exposed macrophages. *Xenobiotica Fate Foreign Compd Biol Syst* 2021;**51**:1436–1452.

27. Kılıç A, Santolini M, Nakano T, Schiller M, Teranishi M, Gellert P et al. A systems immunology approach identifies the collective impact of 5 miRs in Th2 inflammation. *JCI Insight* 2018;**3**:e97503, 97503.

28. Fan Q, Jian Y. MiR-203a-3p regulates TGF-β1-induced epithelial-mesenchymal transition (EMT) in asthma by regulating Smad3 pathway through SIX1. *Biosci Rep* 2020;**40**:BSR20192645.

29. Yu X, Zhe Z, Tang B, Li S, Tang L, Wu Y et al. α-Asarone suppresses the proliferation and migration of ASMCs through targeting the lncRNA-PVT1/miR-203a/E2F3 signal pathway in RSV-infected rats. *Acta Biochim Biophys Sin* 2017;**49**:598–608.

30. Jardim MJ, Dailey L, Silbajoris R, Diaz-Sanchez D. Distinct microRNA expression in human airway cells of asthmatic donors identifies a novel asthma-associated gene. *Am J Respir Cell Mol Biol* 2012;**47**:536–542.

31. Davis JS, Sun M, Kho AT, Moore KG, Sylvia JM, Weiss ST et al. Circulating microRNAs and association with methacholine PC20 in the Childhood Asthma Management Program (CAMP) cohort. *PloS One* 2017;**12**:e0180329.

32. Bahmer T, Krauss-Etschmann S, Buschmann D, Behrends J, Watz H, Kirsten A-M et al. RNA-seq-based profiling of extracellular vesicles in plasma reveals a potential role of miR-122-5p in asthma. *Allergy* 2021;**76**:366–371.

33. Luo X, Zeng Q, Yan S, Liu W, Luo R. MicroRNA-375-mediated regulation of ILC2 cells through TSLP in allergic rhinitis. *World Allergy Organ J* 2020;**13**:100451.

34. Xu G, Xie Q, Zhou H. [Changes of serum miR-375 and blood target genes in patients with allergic rhinitis before and after treatment and its significance]. *Zhong Nan Da Xue Xue Bao Yi Xue Ban* 2019;**44**:767–774.

35. Wang T, Wang P, Chen D, Xu Z, Yang L. circARRDC3 contributes to interleukin‑13‑induced inflammatory cytokine and mucus production in nasal epithelial cells via the miR‑375/KLF4 axis. *Mol Med Rep* 2021;**23**:141.

36. Lu TX, Lim E-J, Wen T, Plassard AJ, Hogan SP, Martin LJ et al. MiR-375 is downregulated in epithelial cells after IL-13 stimulation and regulates an IL-13-induced epithelial transcriptome. *Mucosal Immunol* 2012;**5**:388–396.

37. Wang T, Chen D, Wang P, Xu Z, Li Y. miR-375 prevents nasal mucosa cells from apoptosis and ameliorates allergic rhinitis via inhibiting JAK2/STAT3 pathway. *Biomed Pharmacother Biomedecine Pharmacother* 2018;**103**:621–627.

38. Specjalski K, Niedoszytko M. MicroRNAs: future biomarkers and targets of therapy in asthma? *Curr Opin Pulm Med* 2020;**26**:285–292.

39. Zahm AM, Menard-Katcher C, Benitez AJ, Tsoucas DM, Le Guen CL, Hand NJ et al. Pediatric eosinophilic esophagitis is associated with changes in esophageal microRNAs. *Am J Physiol Gastrointest Liver Physiol* 2014;**307**:G803-812.

40. Lu TX, Sherrill JD, Wen T, Plassard AJ, Besse JA, Abonia JP et al. MicroRNA signature in patients with eosinophilic esophagitis, reversibility with glucocorticoids, and assessment as disease biomarkers. *J Allergy Clin Immunol* 2012;**129**:1064-1075.e9.

41. Beheshti R, Halstead S, McKeone D, Hicks SD. Understanding immunological origins of atopic dermatitis through multi-omic analysis. *Pediatr Allergy Immunol Off Publ Eur Soc Pediatr Allergy Immunol* 2022;**33**:e13817.

42. Simpson MR, Brede G, Johansen J, Johnsen R, Storrø O, Sætrom P et al. Human Breast Milk miRNA, Maternal Probiotic Supplementation and Atopic Dermatitis in Offspring. *PloS One* 2015;**10**:e0143496.

43. Lu TX, Rothenberg ME. Diagnostic, functional, and therapeutic roles of microRNA in allergic diseases. *J Allergy Clin Immunol* 2013;**132**:3–13; quiz 14.

44. Shen Y, Jiang A, Chen R, Gao X, Song G, Lu H. MicroRNA-885-3p alleviates bronchial epithelial cell injury induced by lipopolysaccharide via toll-like receptor 4. *Bioengineered* 2022;**13**:5305–5317.
